# Stability Indicators in Network Reconstruction

**DOI:** 10.1371/journal.pone.0089815

**Published:** 2014-02-27

**Authors:** Michele Filosi, Roberto Visintainer, Samantha Riccadonna, Giuseppe Jurman, Cesare Furlanello

**Affiliations:** 1 MPBA/Center for Information and Communication Technology, Fondazione Bruno Kessler, Trento, Italy; 2 CIBIO, University of Trento, Trento, Italy; 3 Department of Computational Biology, Research and Innovation Centre, Fondazione Edmund Mach (FEM), San Michele all'Adige, Italy; Leibniz-Institute for Farm Animal Biology (FBN), Germany

## Abstract

The number of available algorithms to infer a biological network from a dataset of high-throughput measurements is overwhelming and keeps growing. However, evaluating their performance is unfeasible unless a ‘gold standard’ is available to measure how close the reconstructed network is to the ground truth. One measure of this is the stability of these predictions to data resampling approaches. We introduce NetSI, a family of Network Stability Indicators, to assess quantitatively the stability of a reconstructed network in terms of inference variability due to data subsampling. In order to evaluate network stability, the main NetSI methods use a global/local network metric in combination with a resampling (bootstrap or cross-validation) procedure. In addition, we provide two normalized variability scores over data resampling to measure edge weight stability and node degree stability, and then introduce a stability ranking for edges and nodes. A complete implementation of the NetSI indicators, including the Hamming-Ipsen-Mikhailov (HIM) network distance adopted in this paper is available with the R package *nettools*. We demonstrate the use of the NetSI family by measuring network stability on four datasets against alternative network reconstruction methods. First, the effect of sample size on stability of inferred networks is studied in a gold standard framework on yeast-like data from the Gene Net Weaver simulator. We also consider the impact of varying modularity on a set of structurally different networks (50 nodes, from 2 to 10 modules), and then of complex feature covariance structure, showing the different behaviours of standard reconstruction methods based on Pearson correlation, Maximum Information Coefficient (MIC) and False Discovery Rate (FDR) strategy. Finally, we demonstrate a strong combined effect of different reconstruction methods and phenotype subgroups on a hepatocellular carcinoma miRNA microarray dataset (240 subjects), and we validate the analysis on a second dataset (166 subjects) with good reproducibility.

## Introduction

The problem of inferring a biological network structure given a set of high-throughput measurements, *e.g.* gene expression arrays, has been addressed by a large number of different methods published in the last fifteen years (see [1], [2] for two recent comparative reviews). Solutions range from general purpose algorithms (such as correlation [3] or relevance networks [4]) to methods tailored *ad hoc* for specific data types. Recent examples include SeqSpider [5] for Next Generation Sequencing data, or Sparsity-aware Maximum Likelihood [6] for cis-expression quantitative trait loci (cis-eQTL).

However, network reconstruction is an underdetermined problem, since the number of interactions is significantly larger than the number of independent measurements [7]. Thus, all algorithms must aim to find a compromise between reconstruction accuracy and feasibility: simplifications inevitably detract from the precision of the final outcome by including a relevant number of false positive links [8], which should be discarded *e.g.,* by identifying and removing unwanted indirect relations [9]. Moreover, inference accuracy is strongly dependent on the assumptions used to choose the best hypothetical model of experimental observations [10].

These issues make the inference problem “a daunting task” [11] not only in terms of devising an effective algorithm, but also in terms of quantitatively interpreting the results obtained. In general, reconstruction accuracy is far from optimal in many situations and several pitfalls may occur [12], related to both the methods and the data [13]. In extreme cases, many link predictions are statistically equivalent to random guesses [14]. In particular, it is now widely acknowledged that the size and quality of the data play a critical role in the inference process [15]-[18]. All these considerations support the opinion that network reconstruction should still be regarded as an unsolved problem [19].

Given the growing list of available algorithms, efforts have been made to develop methods for the objective comparison of network inference methods including the identification of current limitations [20], [21] and their relative strengths and disadvantages [7], [22]. The most systematic effort is probably the international DREAM challenge [23]: from DREAM 2012 emerged a consensus advocating the integration of predictions from multiple inference methods as an effective strategy to enhance performance [24]. However, algorithm uncertainty has so far been assessed only in terms of performance, *i.e.*, the distance of the reconstructing network from the ground truth, whenever available, while the stability of the methods has been neglected. When no gold standard is available for a given problem, there is no chance to evaluate algorithm accuracy. In such cases we can consider stability as a rule of thumb for judging the reliability of the resulting network. Obviously, the performance of a network reconstruction algorithm and the stability/reliability of the resulting network inferred from a specific dataset are two distinct and equally crucial aspects of the network inference process. The best way to optimize both aspects would be to adopt only network reconstruction algorithms with well characterized performance, *i.e.,* evaluated in cases where the ground truth is known, and with stability always checked on specific data. It is also worthwhile noting that the evaluation of inference stability is not related to the (chemical or physical) “stability” of the represented process.

We propose to tackle the stability issue by quantifying inference variability with respect to data perturbation, and, in particular, data subsampling. If a portion of data is randomly removed before inferring the network, the resulting graph is likely to be different from the one reconstructed from the whole dataset and, in general, different subsets of data would give rise to different networks. Thus, in the spirit of applying reproducibility principles to this field, one has to accept the compromise that the inferred/non inferred links are just an estimation, lying within a reasonable probability interval. Here we introduce the Network Stability Indicators (NetSI) family, a set of four indicators allowing the researcher to quantitatively evaluate the reproducibility of the reconstruction process. We propose to quantitatively assess, for a given ratio of removed data and for a given amount of (bootstrap [25] or cross-validation) resampling, the mutual distances among all inferred networks and their distances to the network generated by the whole dataset, with the idea that, the smaller the average distance, the more stable the inferred network. Similarly, we propose two indicators for the distribution of variability of the link weight and node degree across the generated networks, providing a ranked list of the most stable links and nodes, the least variable being the top ranked. The described framework for evaluating the stability of the whole network obviously relies on a network distance, but it is independent from the chosen metric. As network distance we use the Hamming-Ipsen-Mikhailov (HIM) distance [26], or its components for demonstration purposes, because it represents a good compromise between local (link-based) and global (structure-based) measures of network comparison. Moreover, the HIM distance can be easily included in pipelines for network analysis [27].

We first show the effect of network modularity and the dataset sample size on both the stability and the accuracy of the network inference process. For this purpose, we create two synthetic datasets with a known gold standard. The results are demonstrated for several inference algorithm, such as the Algorithm for the Reconstruction of Accurate Cellular Networks (ARACNE), developed for the reconstruction of gene regulatory networks [28], the Context Likelihood of Relatedness (CLR) approach [29] and the Weighted Gene Correlation Network Analysis (WGCNA) [30]. Then the NetSI indicators are computed on correlation networks developed on another *ad hoc* synthetic dataset. We highlight the difference in terms of stability due to the choice of the inference algorithm: two basic correlation measures and the impact of a permutation-based False Discovery Rate (FDR) filter. Finally, we show the use of NetSI measures in a typical application, comparing the stability of relevance networks inferred on a miRNA microarray dataset with paired tissues extracted from a cohort of 241 hepatocellular carcinoma patients [31], [32]. The data exhibit two phenotypes, one related to disease (tumoral or non-tumoral tissues) and one to patient gender (male or female); we show that four different networks are obtained, each of different stability, and that the reconstruction method is a serious source of variability with the smaller data subgroups. Finally we validate the analysis on a second hepatacellular carcinoma dataset (166 subjects) with good reproducibility.

All the methods (HIM distance and NetSI indicators) have been implemented in the open source R package *nettools* for the CRAN archives, as well as on GitHub at the address https://github.com/MPBA/nettools.git. For computing efficiency, the software can be used on multicore workstations and on high performance computing (HPC) clusters. Further technical details and preliminary experiments with *nettools* are available in [33].

## Methods

Before defining the NetSI family we briefly summarize the main definitions and properties of the HIM network distance. Moreover, at the end of this section, we provide a short description of the network inference approaches used in the following experiments.

### HIM network distance

The HIM distance [26] is a metric for network comparison combining an edit distance (Hamming [34], [35]) and a spectral one (Ipsen-Mikhailov [36]). As discussed in [37], edit distances are local, *i.e.* they focus only on the portions of the network interested by the differences in the presence/absence of matching links. Spectral distances evaluate instead the global structure of the compared topologies, but they cannot distinguish isomorphic or isospectral graphs, which can correspond to quite different conditions within the biological context. Their combination into the HIM distance represents an effective solution to the quantitative evaluation of network differences.

Let 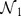 and 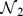 be two simple networks on 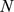 nodes, described by the corresponding adjacency matrices 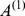 and 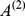, with 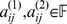, where 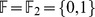 for unweighted graphs and 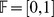 for weighted networks. Denote then by 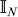 the identity 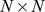 matrix 
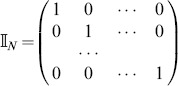
, by 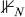 the unitary 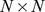 matrix with all entries equal to one and by 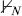 the null 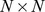 matrix with all entries equal to zero. Finally, denote by 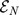 the empty network with 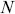 nodes and no links (with adjacency matrix 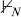) and by 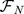 the undirected full network with 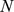 nodes and all possible 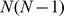 links (whose adjacency matrix is 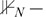 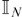).

The definition of the Hamming distance is the following:



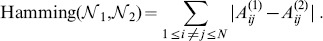



To guarantee independence from the network dimension (number of nodes), we normalize the above function by the factor 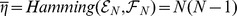:



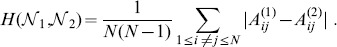
(1)


When 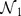 and 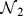 are unweighted networks, 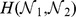 is just the fraction of different matching links (over the total number 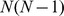 of possible links) between the two graphs. In all cases, 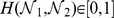, where the lower bound 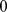 is attained only for identical networks 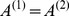 and the upper bound 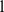 is reached whenever the two networks are complementary 
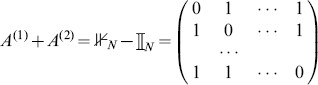
.

Among spectral distances, we consider the Ipsen-Mikhailov distance IM which has been proven to be the most robust in a wide range of situations [37], [38]. Originally introduced in [36] as a tool for network reconstruction from its Laplacian spectrum, the definition of the Ipsen-Mikhailov metric follows the dynamical interpretation of a 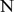
–nodes network as a 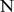
–atoms molecule connected by identical elastic strings, where the pattern of connections is defined by the adjacency matrix of the corresponding network. In particular the connections between nodes in the network correspond to the bonds between atoms in the dynamical system and the adjacency matrix is its topological description.

We summarize here the mathematical details of the IM definition [36]. The dynamical properties of the oscillatory system are described by the set of 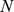 differential equations
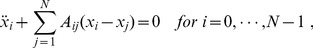
(2)


where 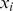 are the coordinates of the physical molecules. Since the adjacency matrix 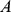 depends on the node labeling, we consider instead the Laplacian matrix 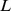, which for an undirected network is defined as the difference between the degree matrix 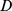 (the diagonal matrix with vertex degrees as entries) and 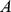: 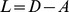. 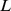 is positive semidefinite and singular [39]–[42], and its set of eigenvalues 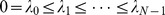, *i.e.* the spectra of the associated graph, provide the natural vibrational frequencies 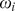 for the system modeled in Eq. 2: 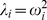, with 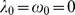. The spectral density 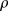 for a graph can be written as the sum of Lorentz distributions
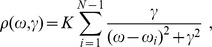



where 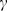 is the common width and 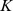 is the normalization constant defined as
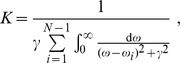



so that 
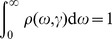
. The scale parameter 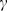 specifies the half-width at half-maximum, which is equal to half the interquartile range. From the above definitions, the spectral distance 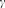 between two graphs 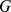 and 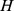 with densities 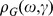 and 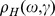 can then be defined as



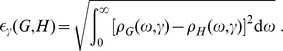



The highest value of 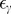 is reached, for a given number of nodes 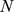, when evaluating the distance between 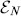 and 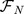. Defining 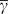 as the (unique [26]) solution of



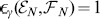



we can now define the normalized Ipsen-Mikahilov distance IM as



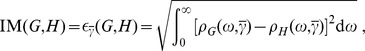



so that 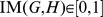 with the upper bound attained only for 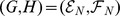.

Finally, the generalized HIM distance is defined by the one-parameter family of product metrics linearly combining with a factor 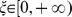 the normalized Hamming distance H and the normalized Ipsen-Mikhailov IM distance, further normalized by the factor 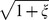 to set its upper bound to 1:
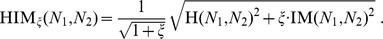



Obviously, 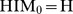 and 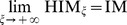. For example, the flexibility introduced by 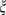 can be used to focus attention more on structure than on local editing changes when 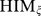 is used to generate a kernel function for classification tasks (*e.g.* on brain networks).

**Figure 1:**
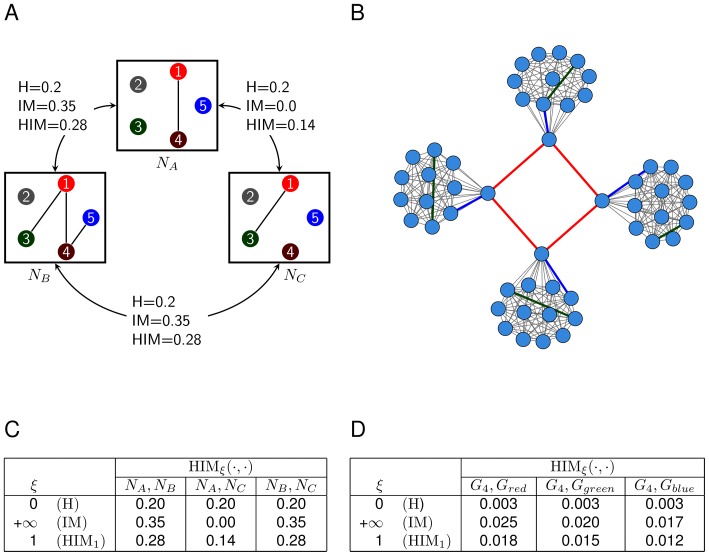
HIM distance: contribution of H and IM. (A) An example on three 5-node networks mutually differing by two links. (B) An example on network 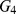, as defined in Subsection *Stability is modularity invariant*. 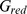: network 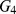 without the four red links. 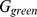: network 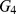 without green links. 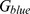: network 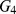 without blue links. (C) The mutual differences between the pairs of networks in (A), 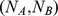 and 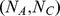. (D) 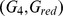, 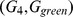, 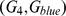. In both cases they have the same Hamming distance but different spectral structure, thus resulting in different Ipsen-Mikhailov distances.

In what follows we will mostly deal with the case 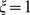, and omit the subscript 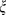 for brevity. The relative effect of the two components is exemplified in Fig. 1A-D. The three small size networks (5 nodes) 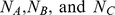 in Fig. 1A differ from each other in only two edges but 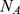 and 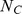 are isomorphic and diverse from 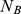, as correctly picked up by the HIM distance (see table in Fig. 1C). Similarly, HIM, H and IM provide different values when four edges are cut from on the larger (50 nodes) 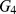 network, at different levels of the graph structure. Larger effects are caused by the elimination of the four red edges connecting the four submodules with differences up to 10 times larger for IM with respect to H (see table in Fig. 1D).

**Figure 2:**
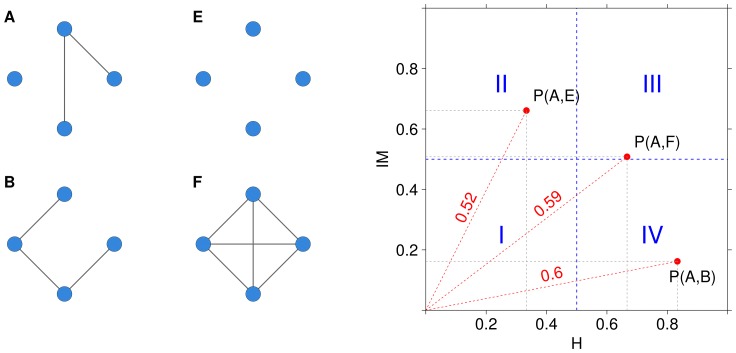
An example of HIM distance. Representation of the HIM distance in the Ipsen-Mikhailov (IM axis) and Hamming (H axis) distance space between networks A versus B, E and F, where E is the empty network and F is the fully connected one.

The HIM distance can be represented in the 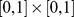 Hamming/Ipsen-Mikhailov space, where a point 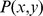 represents the distance between two networks 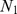 and 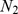 whose coordinates are 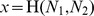 and 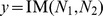 and the norm of 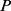 is 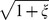 times the HIM distance 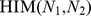. The same holds for weighted networks, provided that the weights range over 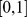. In Fig. 2 we provide an example of this representation by evaluating the HIM distance between networks of four nodes, namely networks A, B, E (empty) and F (full) in the left panel of Fig. 2. If the Hamming/Ipsen-Mikhailov space is roughly split into four quadrants I, II, III, and IV, then two networks whose distance is mapped in quadrant I are close both in terms of matching links and of structure, while those falling in quadrant III differ with respect to both characteristics. Networks corresponding to a point in quadrant II have many common links, but different structures, while a point in quadrant IV indicates two networks with few common links, but with similar structure.

Full mathematical details about the HIM distance and its two components H and IM can be found in [26].

### The Network Stability Indicators (NetSI)

**Figure 3:**
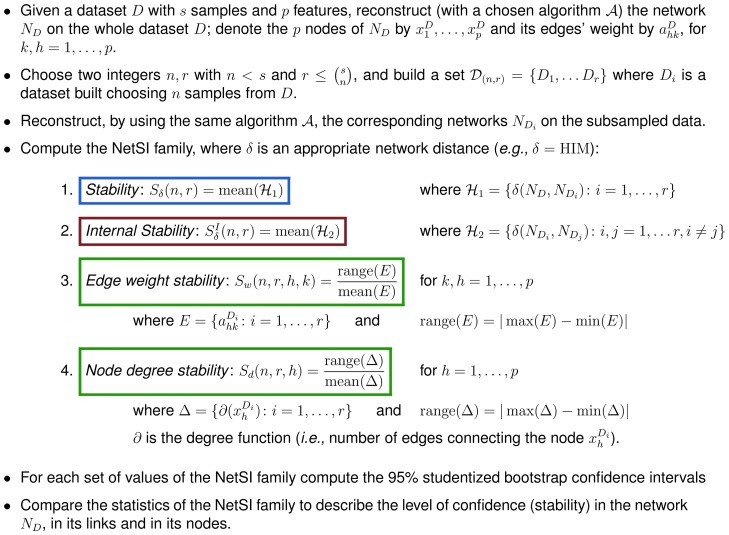
Definition of the NetSI family.

The mathematical and operational definition of the four NetSI indicators are introduced in Fig. 3. The first two are the stability indicators 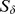 and the internal stability indicator 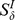, which concern the stability of the whole reconstructed network. The former measures the distances between the network inferred on the whole dataset against the networks inferred from the resampled subsets. The latter measures all the mutual distances within the networks inferred from the resampled subsets. The other two indicators, the edge weight stability indicator 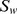 and the node degree stability indicator 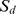, concern instead the stability of the single links and nodes, in terms of mutual variability of their respective weight and degree. In all cases, smaller indicator values correspond to more stable objects.

**Figure 4:**
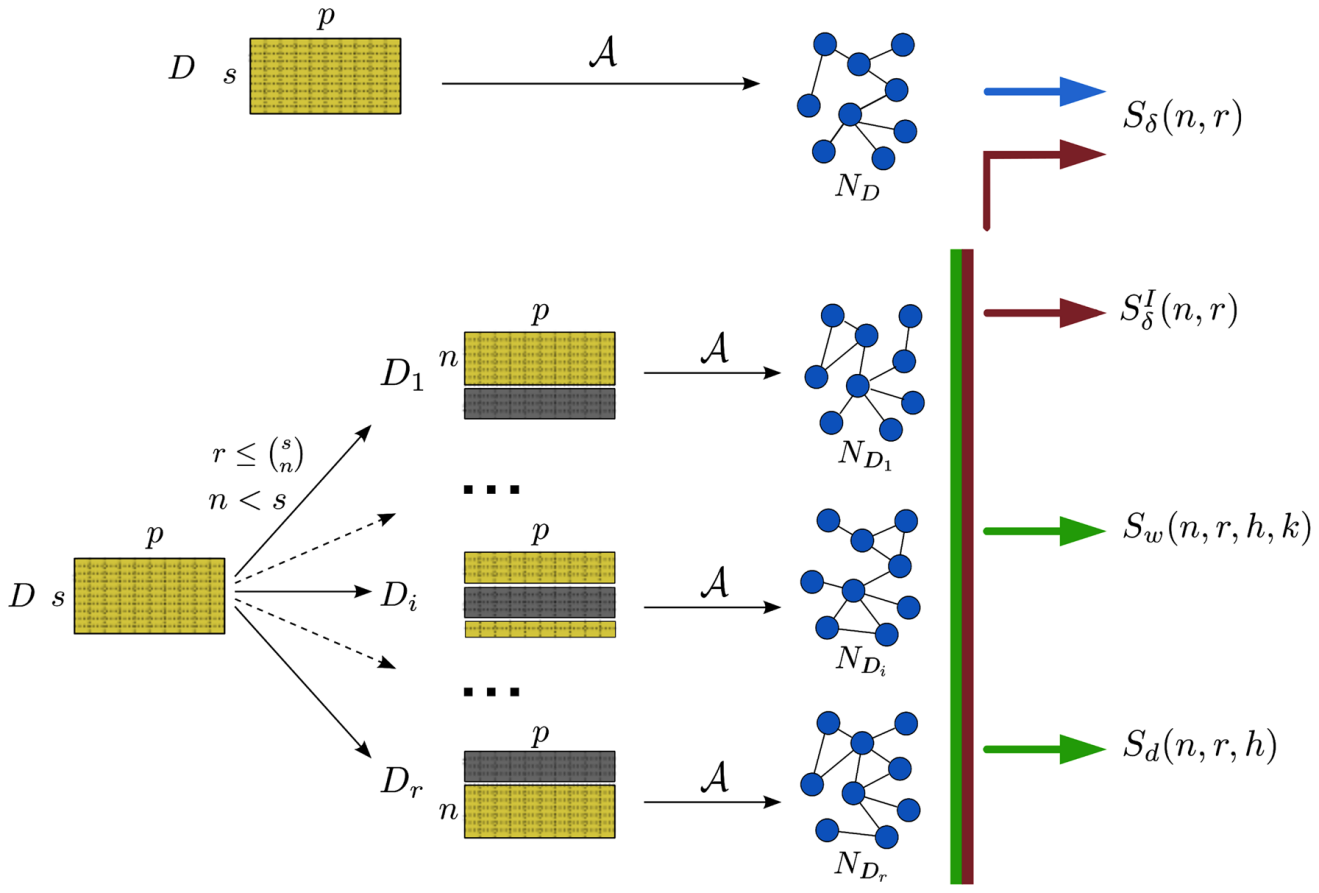
Graphical description of the pipeline in Fig. 3. Using the inference algorithm 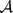, the network 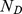 is first reconstructed from the whole dataset 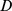 with 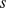 samples and 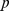 features (nodes). Given two integers 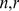, a set of 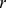 datasets 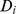 is generated by choosing for each 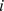 a subset of 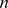 samples from 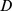, and the corresponding networks 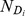 are inferred by 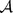. Finally, the four indicators 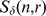, 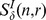, 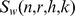 and 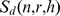 are computed according to their definition.

We adopt 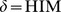, except for the first experiment where we show also the stability for 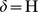 and 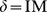. As the HIM distance is defined also on directed networks, the extension of the NetSI family to the directed case is straightforward. A graphical representation of the procedure is provided in Fig. 4. For all experiments reported in this paper, we used 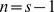, 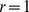 (leave-one-out stability, LOO for short), and 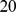 different instances of 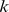-fold cross validation (discarding the test portion) for 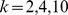 (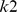, 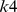 and 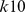), and thus 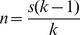 and 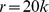.

### Stability of network inference algorithms

As a first application, we test the difference in stability of the reconstruction process for a set of alternative network co-expression inference algorithms.

The most famous representative of the correlation-based approaches is surely the Weighted Gene Correlation Network Analysis (WGCNA) [30], [43]. In this case the co-expression similarity is defined as a function of the absolute correlation. We adopt as similarity score: (i) the simple absolute Pearson correlation (labelled as “cor”), (ii) a more sophisticated version with soft-thresholding, *i.e.*, the similarity is defined as a power of the absolute correlation (we adopt the default value six as in the *WGCNA* R package), or (iii) the biweight midcorrelation (“bicor” for short) [30], [44], which is more robust to outliers than the Pearson correlation, and (iv) the Maximal Information Coefficient (labeled as MIC). MIC is a recent association measure based on mutual information and belongs to the Maximal Information-based Nonparametric Exploration (MINE) statistics [44]–[48]. In all cases we obtain a weighted network with link strength ranging from 0 to 1.

The Topological Overlap Measure (TOM) replaces the original co-expression similarity with a measure of interconnectedness (between pairs of nodes) based on shared neighbors [30], [43]. TOM can be seen as a filter for cutting away weak connections, thus leading to more robust networks than WGCNA.

The Context Likelihood of Relatedness (CLR) approach [29] scores the interactions by using the mutual information between the corresponding gene expression levels, coupled with an adaptive background correction step. Although suboptimal if the number of nodes is much larger than the number of variables, it was observed that CLR performs well in terms of prediction accuracy and some CLR predictions in literature were recently validated experimentally [49].

The Algorithm for the Reconstruction of Accurate Cellular Networks (ARACNE) is another approach relying on mutual information, which was originally developed for inferring regulatory networks of mammalian cells [28]. It starts with a graph where each pair of nodes are connected if their association is above a chosen threshold. In order to avoid the false positive problem, that usually affects co-expression networks, we then apply the Data Processing Inequality (DPI) procedure for removing the weakest edge of each triplet, thus pruning the majority of undirected links.

A unique interface to all the mentioned algorithms is integrated in the stability analysis tools in the *nettools* package, based on their Bioconductor and CRAN implementations: *minet* for ARACNE and CLR, *WGCNA* for WGCNA, TOM and bicor, and *minerva* for MIC.

## Results and Discussion

### Stability is modularity invariant

We demonstrate the invariance of the NetSI family with respect to network modularity in a controlled situation. We show that the proposed stability evaluation framework is not affected by various network structures for nine reconstruction algorithms. Moreover, we demonstrate that this property is maintained both if we adopt the HIM metric for the 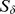 indicator computation and we use the two components H and IM separately.

#### Data generation

**Figure 5:**
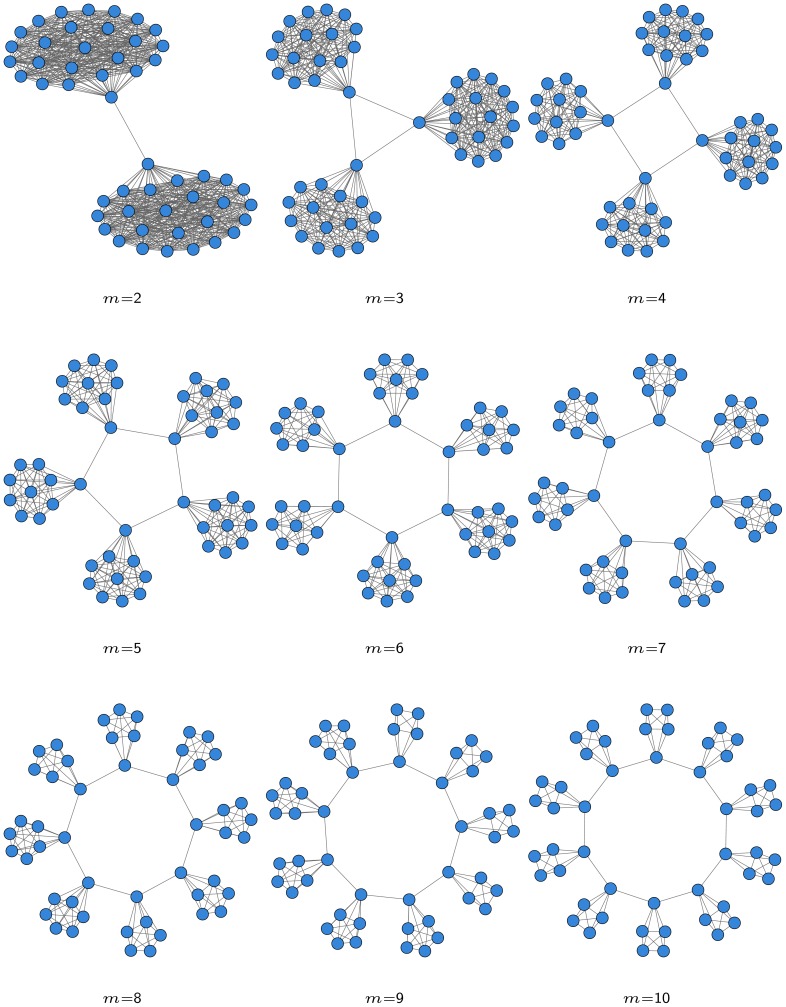
Synthetic network with 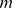 modules, where 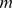 ranges from 2 to 10 from top left to bottom right.

We created a set of networks 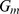 with 50 nodes each with 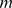 (where 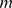 ranges from 1 to 10) fully connected subgroups, which are linked to each other with a single edge. For 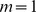 we obtain a fully connected network (without loops), while the resulting networks for 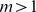 are displayed in Fig. 5. For each network 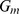 we report its modularity value and density in Tab. 1.

**Table 1:** Modularity and density values for 50-nodes networks (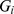) for an increasing number of modules 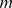.

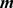	Modularity	Density
1	0.00	1.00
2	0.50	0.49
3	0.66	0.32
4	0.73	0.24
5	0.78	0.19
6	0.80	0.16
7	0.81	0.13
8	0.82	0.11
9	0.81	0.10
10	0.81	0.09

The simulated gene expression values corresponding to the networks 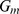 are generated loading the corresponding adjacency matrices in the Gene Net Weaver (GNW) simulator [50]. Specifically, the tool is used to create of simulated transcription datasets after a random initialization of each network's regulatory dynamics through a pre-loaded kinetic model [23]. Moreover it is possible to generate a steady-state dataset or a set of time series, which describes the network response to a perturbation, followed by perturbation removal until the steady state is reached. Thus, we chose to generate in one shot 50 time-series (one for each sample) with default parameter settings and to consider only the initial time point, since 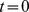 corresponds to the wild-type steady state. Summarizing, we generated 10 synthetic datasets having a simulated expression level for 50 “genes” and 50 “samples”.

#### Results

We inferred networks from the 10 datasets with nine algorithms: ARACNE, CLR, cor, TOM, bicor, WGCNA and MIC, where the last two were also used with a permutation-based FDR filter (for details, see Subsection “FDR control effect on correlation networks”).

**Figure 6:**
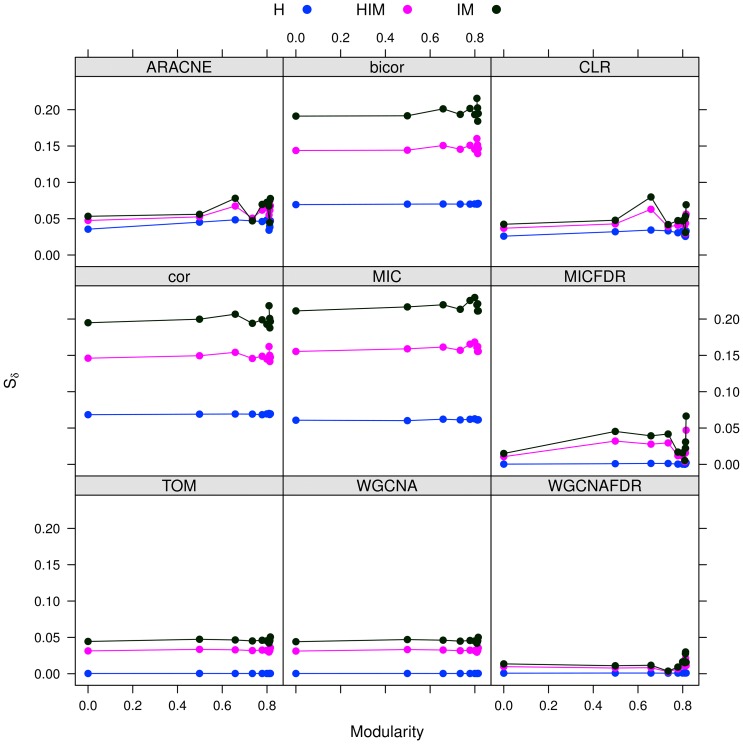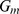 networks: Stability of synthetic networks for different modularity levels.

**Figure 7:**
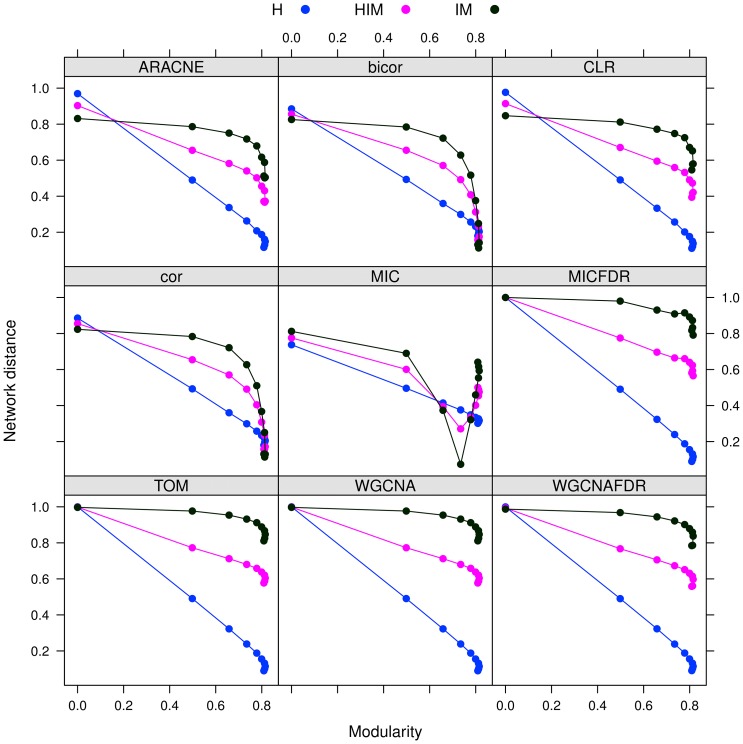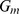 networks: distance between gold standard (HIM) and inferred synthetic networks for different modularity levels.

The stability analysis with three possible network metrics (HIM, H and IM) on networks inferred with the nine mentioned approaches is reported in Fig. 6. In all cases, the stability 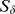 varies less than 0.06 across different modularity values, as detailed in Tab. 2. Hence, the stability indicator is not affected by different modular structures. However, reconstruction accuracy depends on modularity (or density), as shown by a comparison with the gold standard (Fig. 7), in which a lower distance from the gold standard is found for sparser networks for all methods.

**Table 2:** 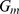
 networks: range of 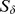 for different reconstruction algorithms and 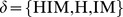.

	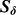 variation range		
algorithm	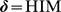	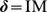	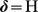
WGCNA	0.01	0.01	0.00
WGCNAFDR	0.02	0.03	0.00
cor	0.02	0.03	0.00
MIC	0.01	0.02	0.00
MICFDR	0.04	0.06	0.00
ARACNE	0.02	0.03	0.01
bicor	0.02	0.03	0.00
TOM	0.01	0.01	0.00
CLR	0.03	0.05	0.01

### Inference on synthetic yeast-like networks

We investigated the behavior of the NetSI stability indicators for different sample sizes on a yeast-like dataset, again simulated by GNW.

#### Data Description

We considered a subnetwork of the Yeast transcriptional regulatory network available in GNW, namely the *InSilicoSize100-Yeast2* dataset with 100 nodes, originally a DREAM3 benchmark, generating 100 samples with default parameter configuration, including noise level, for wild-type steady state (the synthetic dataset 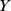).

#### Results

We randomly extracted 10 subsets of different sample size 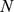 in 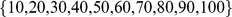, replicating the subset extraction procedure 50 times for each 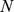. For each combination of 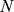 resampling, we inferred the network with the same nine algorithms used in the previous experiment.

**Figure 8:**
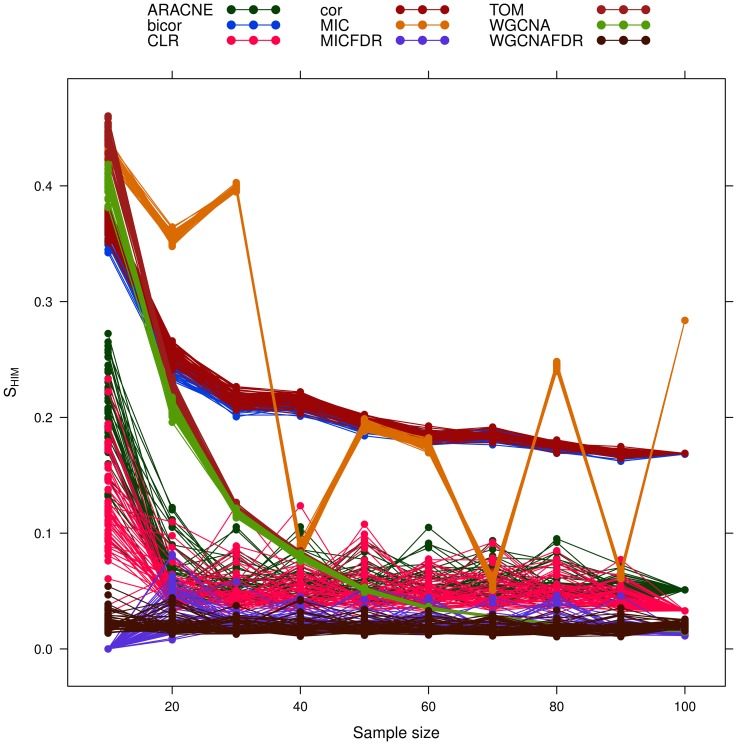
Yeast-like simulated data: effect of increasing sample size on network reconstruction stability 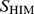. Different network inference algorithms are compared.

**Figure 9:**
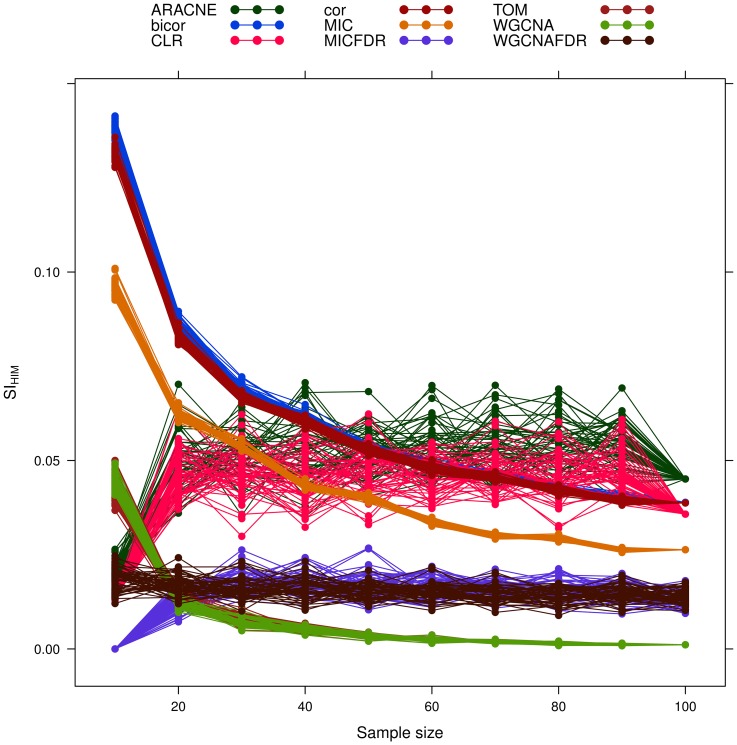
Yeast-like simulated data: effect of increasing sample size on network reconstruction internal stability 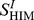. Different network inference algorithms are compared.

As a general trend, stability decreases for larger sample size (see Fig. 8). The 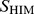 stability curves for the two popular methods ARACNE and CLR drop quickly after 20% of the sample size, improving over Pearson and bicor. TOM and WGCNA are more stable but require at least 50% of the data. The standard MIC-based method with the default parameter (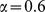) is much smoothed by the FDR correction. Overall, the FDR corrected methods are the most stable even for small samples. TOM and WGCNA have the best internal stability 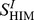 (Fig. 9), followed by the FDR-corrected methods.

**Figure 10:**
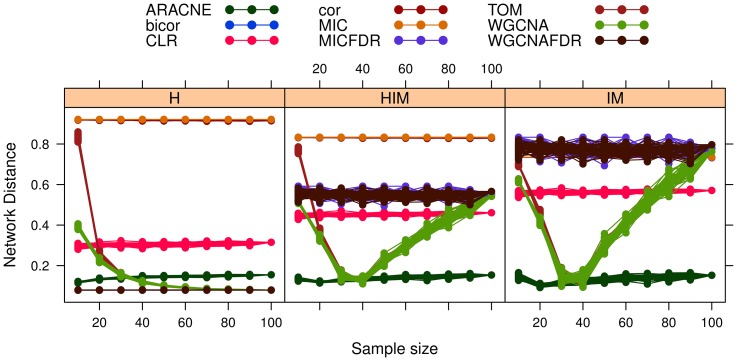
Yeast-like simulated data: effect of increasing sample size on network reconstruction accuracy measured as HIM distance and its components Hamming (H) and Ipsen-Mikhailov (IM) with respect to the gold standard. Different network inference algorithms are compared.

Given that a gold standard is available for the simulated data in 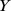, we can compare the stability performance with reconstruction accuracy in terms of HIM and its components for the nine methods (Fig. 10). On this dataset, accuracy is independent of sample size, except for WGCNA and TOM which have an optimal range (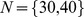), given by their soft thresholding procedures. Note that the source Yeast subnetwork is unweighted while all methods return a weighted network: a 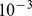 threshold was thus applied to binarize the reconstructed network before computing the distances. Hence, MIC, cor and bicor perform badly as they lack an internal thresholding procedure; the FDR corrected methods have better but still mediocre results, slightly improved by CLR. On this dataset, WGCNA-FDR yields sparse networks (less than 20 edges) with small Hamming distances from the gold standard as they both have low density; however they have strongly different spectral structure from the gold standard, as captured by the IM component. Finally, ARACNE achieves fair stability here as well as the highest accuracy.

### The effect of FDR control on stability

We aim to assess differences in the stability of correlation networks inferred by an *ad-hoc* set of synthetic signals similar to expression data whenever inference is computed with or without False Discovery Rate (FDR) control.

#### FDR control for correlation networks

**Figure 11:**
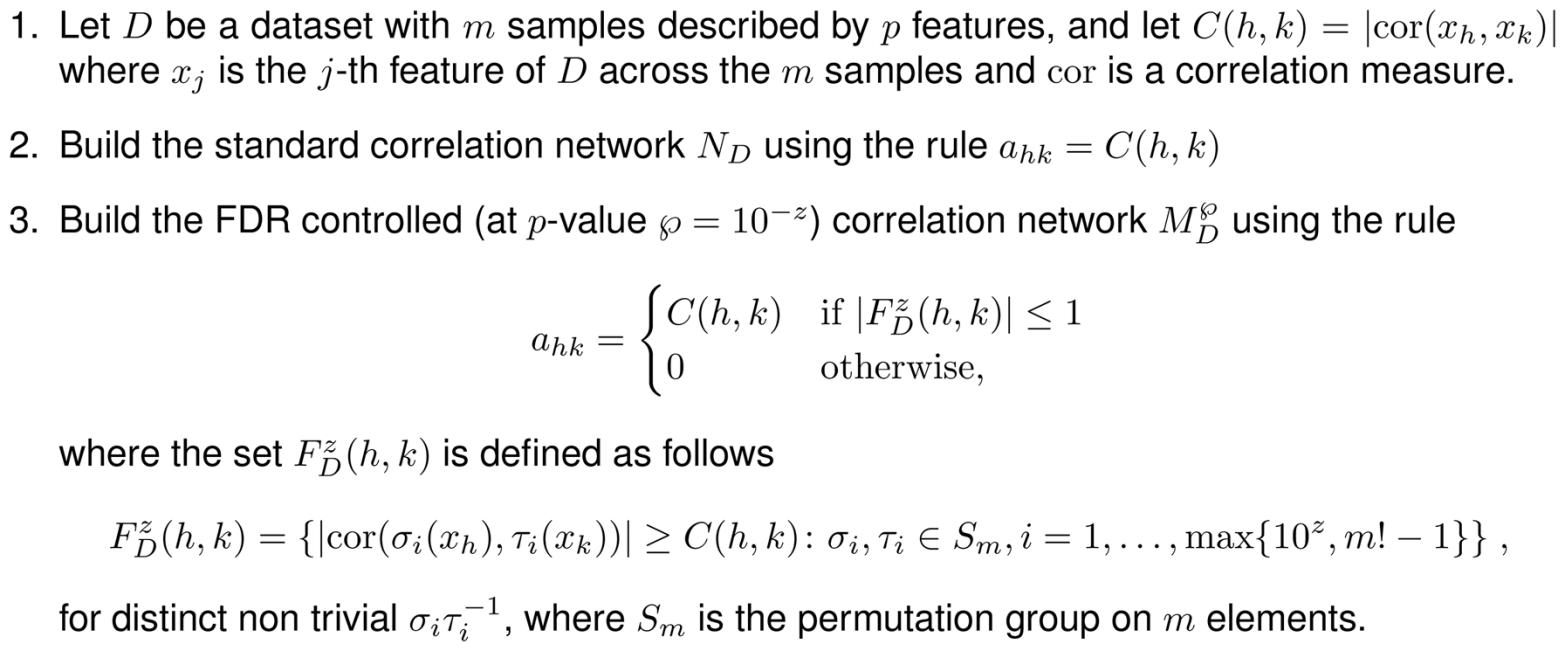
Construction of an FDR-corrected correlation network.

For an introduction to FDR methods, see for instance [51]. The procedure considered in this paper is explicitly described in Fig. 11. The FDR control defines a rule for choosing which edges to trust, and thus to keep, during the network reconstruction phase. An edge weight is given by the correlation coefficient between the signal values of two nodes 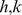: 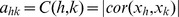, where 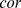 is a correlation function. If 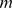 is the sample size, we wish to estimate the chance that a random permutation of the expression values may give a correlation value higher than 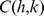, thus removing the edge when this chance is larger than a permutational p-value 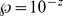, where 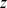 is a chosen level of significance (typically 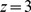). In practice, this test is implemented by counting how many times 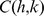 is smaller than the correlation between 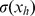 and 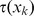, where 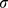 and 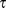 are distinct permutations of 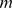 objects. We consider here the absolute Pearson correlation at different 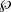 levels CORFDR(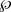), when compared with WGCNA [30], [43] with default thresholding parameter, as well as with the Maximal Information Coefficient (MIC), a non-linear correlation measure defined within the Maximal Information-based Nonparametric Exploration (MINE) statistics [45]–[47]. Note that the FDR correction procedure can be implemented with different correlation measures, such as WGCNA-FDR and MIC-FDR considered in the previous section.

#### Data generation

**Figure 12:**
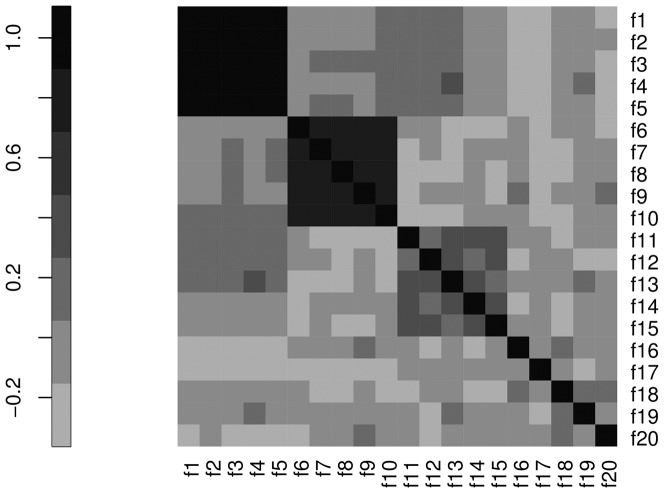
The correlation matrix 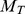 used to generate the synthetic dataset 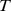.

In this example, the correlation networks are inferred from a dataset 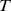 of 100 samples of 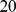 features 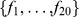, via the Choleski decomposition (using the chol R function) of its correlation matrix 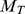, randomly generated according to the following three constraints:



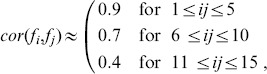



where 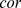 is the Pearson correlation. The correlation matrix 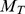 is plotted in Fig. 12: the correlation values in the three groups defined by the above constraints represent true relations between the variables, while all other smaller correlation values are due to the underlying random generation model for 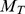.

#### Results

Starting from the dataset 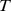, we built five correlation networks, using MIC, WGCNA, and CORFDR(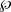) with 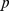-values 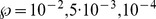.

**Figure 13:**
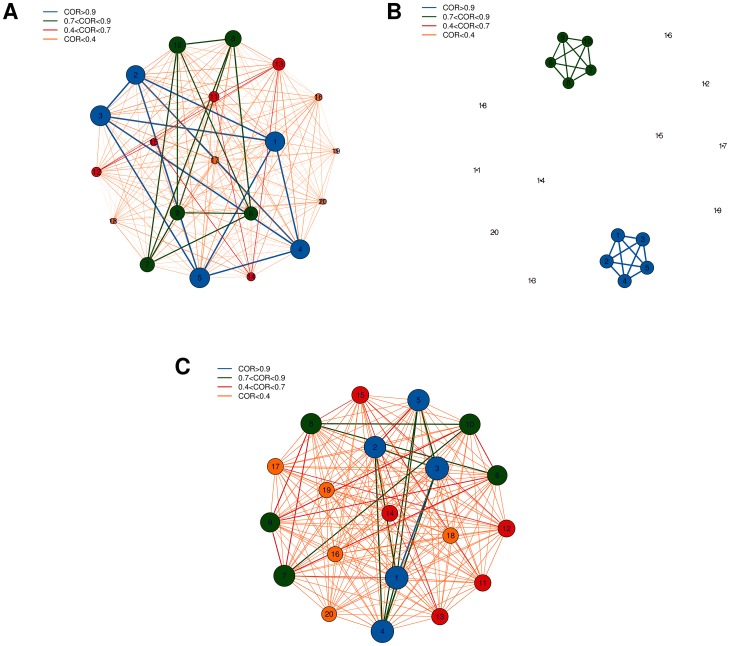
Synthetic dataset 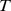: correlation networks inferred by using (A) WGCNA [W], (B) (absolute) Pearson with FDR correction at 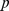-value 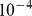 [C(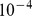)] and (C) MIC [M]. Node label 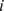 corresponds to feature 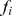, node size is proportional to node degree and link colors identify different classes of link weights.

The three networks displayed in Fig. 13(A-C) were inferred with WGCNA, CORFDR(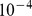) and MIC respectively. WGCNA and MIC generate two fully connected networks with a majority of weak links, while CORFDR correctly selects only links within the two disjoint sets of nodes 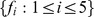 and 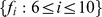, corresponding to the strongest correlations in the matrix 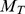.

Note that although both WGCNA and CORFDR(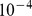) employ 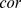 internally, the two algorithms can lead to different results. Indeed the soft thresholding procedure in WGCNA [43] defines weights as 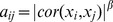 for 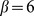, while for CORFDR(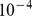) is 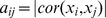 if 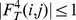, according to the definition in Fig. 11.

**Figure 14:**
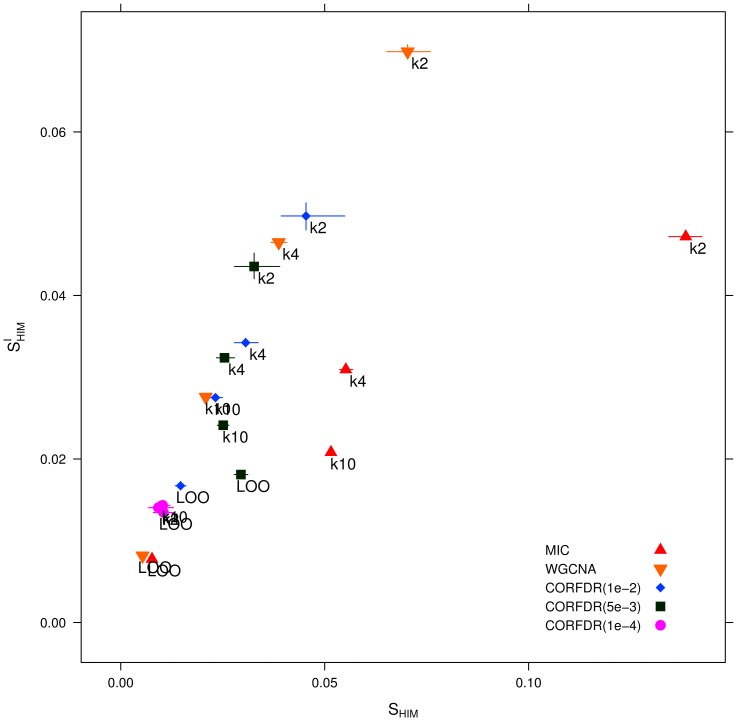
Synthetic dataset 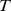: representation of 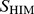 and 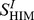 stability indicators (with confidence intervals) for different instances of the FDR-corrected correlation networks, CORFDR(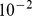), CORFDR(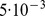), and CORFDR(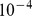), WGCNA and MIC networks on the dataset 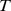 and for different values of data subsampling.

Stability estimates are also less variable with the FDR corrected methods. We compared 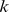-fold cross-validation estimates of 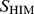 and 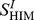 for the five networks, with 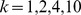. Results are presented in Tab. 3 and displayed in Fig. 14. On this dataset, 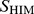 ranges over 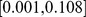 for WGCNA, 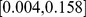 for MIC, and only over 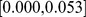 for CORFDR(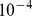). Note that estimates are both smaller and less variable for the smallest p-value, as noise effects are filtered. Hence, the use of a FDR control procedure helps stabilize the correlation based inference procedure, improving the performance of WGCNA, already one of the more robust options for real data [52].

**Table 3:** Synthetic dataset 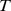: stability (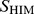 and 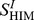) on networks inferred by MIC, WGCNA, and CORFDR(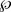).

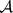	k	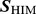	CI (min; max)	Range (min; max)	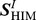	CI (min; max)	Range (min; max)
MIC	LOO	0.008	(0.007; 0.008)	(0.004; 0.011)	0.008	(0.008; 0.008)	(0.003; 0.014)
MIC	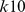	0.052	(0.051; 0.052)	(0.041; 0.067)	0.021	(0.021; 0.021)	(0.014; 0.036)
MIC	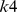	0.055	(0.054; 0.057)	(0.040; 0.071)	0.031	(0.031; 0.031)	(0.022; 0.045)
MIC	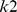	0.139	(0.134; 0.142)	(0.112; 0.158)	0.047	(0.047; 0.048)	(0.035; 0.067)
WGCNA	LOO	0.005	(0.005; 0.006)	(0.001; 0.015)	0.008	(0.008; 0.008)	(0.002; 0.023)
WGCNA	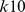	0.021	(0.020; 0.022)	(0.011; 0.040)	0.028	(0.028; 0.028)	(0.012; 0.064)
WGCNA	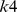	0.039	(0.037; 0.041)	(0.020; 0.062)	0.046	(0.046; 0.047)	(0.025; 0.088)
WGCNA	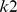	0.070	(0.065; 0.076)	(0.037; 0.108)	0.070	(0.069; 0.071)	(0.042; 0.117)
CORFDR( 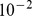 )	LOO	0.015	(0.013; 0.016)	(0.005; 0.035)	0.017	(0.017; 0.017)	(0.001; 0.047)
CORFDR( 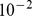 )	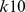	0.023	(0.022; 0.025)	(0.007; 0.074)	0.028	(0.027; 0.028)	(0.002; 0.102)
CORFDR( 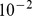 )	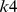	0.031	(0.028; 0.034)	(0.010; 0.069)	0.034	(0.034; 0.035)	(0.006; 0.096)
CORFDR( 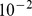 )	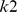	0.045	(0.039; 0.054)	(0.014; 0.107)	0.050	(0.048; 0.051)	(0.006; 0.152)
CORFDR( 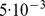 )	LOO	0.029	(0.028; 0.031)	(0.003; 0.048)	0.018	(0.018; 0.018)	(0.000; 0.054)
CORFDR( 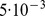 )	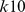	0.025	(0.024; 0.027)	(0.004; 0.054)	0.024	(0.024; 0.024)	(0.001; 0.083)
CORFDR( 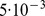 )	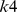	0.025	(0.023; 0.028)	(0.006; 0.056)	0.032	(0.032; 0.033)	(0.004; 0.099)
CORFDR( 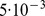 )	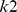	0.033	(0.028; 0.038)	(0.008; 0.070)	0.044	(0.042; 0.045)	(0.002; 0.121)
CORFDR( 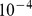 )	LOO	0.010	(0.008; 0.013)	(0.000; 0.044)	0.013	(0.013; 0.014)	(0.000; 0.045)
CORFDR( 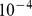 )	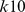	0.010	(0.009; 0.012)	(0.000; 0.053)	0.014	(0.014; 0.015)	(0.000; 0.055)
CORFDR( 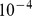 )	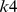	0.009	(0.007; 0.012)	(0.001; 0.049)	0.014	(0.014; 0.014)	(0.001; 0.054)
CORFDR( 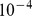 )	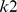	0.009	(0.007; 0.013)	(0.001; 0.031)	0.014	(0.013; 0.015)	(0.001; 0.040)

Indicators 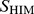 and 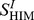, 95% Student bootstrap confidence intervals and range for different instances of the MIC, WGCNA and CORFDR(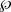) networks for different values of data subsampling.

Results for weight stability and degree stability are listed in Tab. 4 and Tab. 5, respectively, comparing the rankings for WGCNA, CORFDR(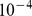) and MIC for a 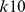 resampling. The stability indicators give information consistent with the structure of the starting correlation matrix 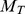: inference by WGCNA (Fig. 13 A) exactly reconstructs the block structure with three subnetworks (blue: 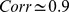, green 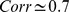, orange: 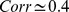), while the FDR corrected version (Fig. 13 B) selects only two subnetworks corresponding to the two top correlated feature blocks of Fig. 12. In the WGCNA case, the top 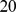 most stable links (Tab. 4) are those of the two cliques 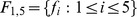 and 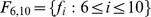 with largest correlation values in 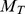. The correct block structure is found by CORFDR(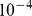) with approximately the same values of 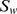 found by the WGCNA network, where minor differences are due to the different thresholding procedures.

**Table 4:** Synthetic dataset 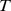: top ranked links for edge weight stability 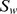 on networks inferred by WGCNA, CORFDR(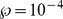), and MIC.

WGCNA		CORFDR( 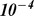 )		MIC	
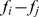	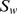	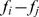	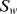	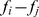	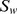
1 – 3	0.03	1 – 3	0.03	3 – 4	0.20
2 – 3	0.04	3 – 4	0.04	2 – 3	0.20
1 – 2	0.04	2 – 3	0.04	1 – 3	0.21
1 – 4	0.04	1 – 4	0.05	3 – 5	0.22
3 – 4	0.04	3 – 5	0.05	1 – 2	0.23
2 – 4	0.04	1 – 2	0.05	1 – 5	0.25
4 – 5	0.04	2 – 4	0.05	1 – 4	0.26
2 – 5	0.05	2 – 5	0.06	4 – 5	0.27
1 – 5	0.05	4 – 5	0.06	7 – 10	0.28
3 – 5	0.05	1 – 5	0.06	7 – 8	0.29
6 – 8	0.08	6 – 8	0.08	6 – 8	0.29
8 – 10	0.10	7 – 8	0.09	6 – 10	0.30
7 – 8	0.11	8 – 10	0.10	1 – 20	0.31
7 – 9	0.11	8 – 9	0.11	2 – 4	0.31
8 – 9	0.11	6 – 7	0.11	8 – 10	0.31
9 – 10	0.11	7 – 10	0.12	2 – 5	0.32
6 – 7	0.11	7 – 9	0.12	9 – 10	0.32
7 – 10	0.12	9 – 10	0.13	7 – 20	0.33
6 – 10	0.13	6 – 9	0.13	14 – 16	0.33
6 – 9	0.14	6 – 10	0.15	5 – 17	0.35
11 – 13	0.33			6 – 7	0.35
14 – 15	0.41			11 – 17	0.36
13 – 14	0.46			6 – 9	0.36
12 – 13	0.58			1 – 10	0.37
12 – 15	0.60			10 – 11	0.37
11 – 14	0.62			10 – 20	0.37
13 – 15	0.71			4 – 17	0.37
11 – 15	0.78			2 – 8	0.37
14 – 18	0.78			4 – 10	0.37
3 – 11	0.83			6 – 13	0.37
5 – 11	0.83			2 – 14	0.37
1 – 11	0.84			9 – 11	0.38
4 – 11	0.85			15 – 16	0.38
3 – 10	0.87			15 – 17	0.38
5 – 16	0.89			7 – 13	0.39
8 – 17	0.89			9 – 18	0.39
2 – 11	0.91			12 – 19	0.39
8 – 12	0.91			6 – 18	0.39
4 – 13	0.91			8 – 9	0.39
1 – 13	0.93			4 – 18	0.39
3 – 13	0.93			16 – 17	0.39
8 – 13	0.94			4 – 19	0.39
9 – 17	0.94			16 – 19	0.39
1 – 16	0.95			7 – 19	0.40
1 – 10	0.95			5 – 8	0.40
14 – 16	0.97			14 – 15	0.40
5 – 10	0.97			13 – 15	0.40
11 – 12	0.98			4 – 11	0.40
12 – 16	0.98			7 – 9	0.41
2 – 13	0.99			13 – 19	0.41

The links are ordered by 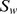 across all 20 resamplings of 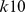 cross validation, for the three algorithms; the table includes the top 50 links for WGCNA and MIC, and all 20 links found by CORFDR(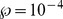).

**Table 5:** Synthetic dataset 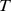: nodes ranked by stability degree 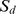 on networks inferred by WGCNA, CORFDR(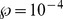), and MIC.

WGCNA		CORFDR( 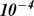 )		MIC	
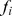	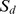	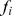	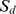	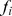	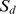
4	0.17	16	0*	3	0.08
10	0.18	17	0*	19	0.08
3	0.20	18	0*	1	0.08
1	0.21	19	0*	4	0.09
9	0.23	20	0*	8	0.09
2	0.23	3	0.03	10	0.09
5	0.24	1	0.04	5	0.10
7	0.24	2	0.04	2	0.10
6	0.24	5	0.05	17	0.10
8	0.25	7	0.07	20	0.10
11	0.40	8	0.07	15	0.11
13	0.40	6	0.09	9	0.11
15	0.43	9	0.09	13	0.11
12	0.45	10	0.09	11	0.11
14	0.48	4	0.13	16	0.11
18	0.55	15	4.42	12	0.11
16	0.60	14	7.05	7	0.11
17	0.68	12	22.82	6	0.12
20	0.70	13	26.05	14	0.13
19	1.15	11	41.83	18	0.13

The 20 nodes are ordered by 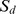 across all 20 resamplings by 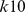 cross validation. (*) indicates that range and mean are both zero.

The 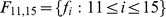 variables (mutual correlation of about 0.3 imposed by design of 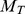) are also mostly top ranked links for WGCNA, but with larger instability values (0.33–0.78 vs. 0.03–0.14). The remaining links are the least stable, with 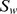 values always larger than 0.83: they are the randomly correlated links of 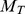. Similar but not identical results are found for the network 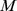, as expected given that the MIC statistic aims at detecting generic associations between variables and it is expected to have reduced statistical power with low sample sizes. The structure of the network (Fig. 13 C) does not reflect the design linear correlation structure. Indeed, several links are ranked differently as the expected: although many links in the 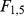 and 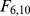 groups are highly ranked, some of them can also be found in much lower positions (*e.g*. 6–7, 6–9, or 7–9 are ranked lower than 20; 7–10, 7–8 are ranked higher than 2–4, 2–5; 1–20, 7–20, 14–16, and 5–17 are ranked within the top 20 links).

Similar considerations hold for the ranking of the most stable nodes: for WGCNA, the top-ranked nodes are the 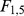 and the 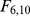 (with similar 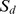 values); those in 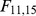 come next, leaving the remaining five as the least stable with higher 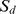 values. These nodes are trivially the most stable for CORFDR(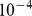) as they are never wired to any other node in any of the resampling and thus their 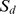 values are void.

The nodes 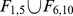 then follow in the ranking with small associated values, and the nodes 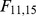 close the standing with definitely higher values. In fact, although the nodes 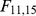 have degree zero in the network CORFDR(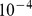) inferred from the whole 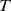, some links connecting them have weight over the threshold in several resamplings. Note that the ranking values for MIC span a narrow range, with most of the nodes in 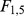 in top positions, in general yielding a weak relation with the structure of 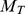.

The weight and degree stability analysis for the other subsampling cases (LOO, 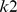 and 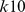) are almost identical and thus not shown here.

### miRNA networks for hepatocellular carcinoma

Investigating the relationships connecting human microRNA (miRNA) and how they evolve in cancer is a key issue for researchers in biology [53], [54]. Hepatocellular carcinoma (HCC) is a notable example [55], [56]: we test the NetSI indicators on a miRNA microarray hepatocellular carcinoma dataset with two phenotypes as a tool for differential network analysis. As CLR was used in the original paper, we applied this inference method and compared its stability with the reconstruction algorithms previously employed on the synthetic datasets.

#### Data description

The HCC dataset (HCC-B) [31], [32] is publicly available at the Gene Expression Omnibus (GEO) http://www.ncbi.nlm.nih.gov/geo with accession number GSE6857. The dataset collects 482 tissue samples from 241 patients affected by HCC. For each patient, a sample from cancerous hepatic tissue and a sample from surrounding non-cancerous hepatic tissue are available, hybridized on the Ohio State University CCC MicroRNA Microarray Version 2.0 platform consisting of 11,520 probes measuring the expression of 250 non-redundant human and 200 mouse miRNAs. After imputation of missing values [57], probes corresponding to non-human (mouse and controls) miRNAs were discarded; samples for one patient (AN) were eliminated. We thus obtained a dataset of 240+240 paired samples described by 210 human miRNAs (210 males, 30 females). Thus HCC-B can be split into four subsets by combining the gender and disease status phenotypes, respectively for tissues of male cancer patients (MT), female cancer patients (FT) and the corresponding non cancer tissues (MnT and FnT).

For validation, we considered a second dataset (HCC-W) recently used to derive a signature of 30 miRNAs for hepatocellular carcinoma [58]. miRNA expression data for 166 subjects (paired samples for 141 males and 25 females), acquired with the CapitalBio custom two-channel microarray platform (692 probes), are available at GEO accession number GSE31384. Data are processed as normalized differential miRNA expression levels between tumors and non cancerous liver tissue data [58].

#### Results

**Figure 15:**
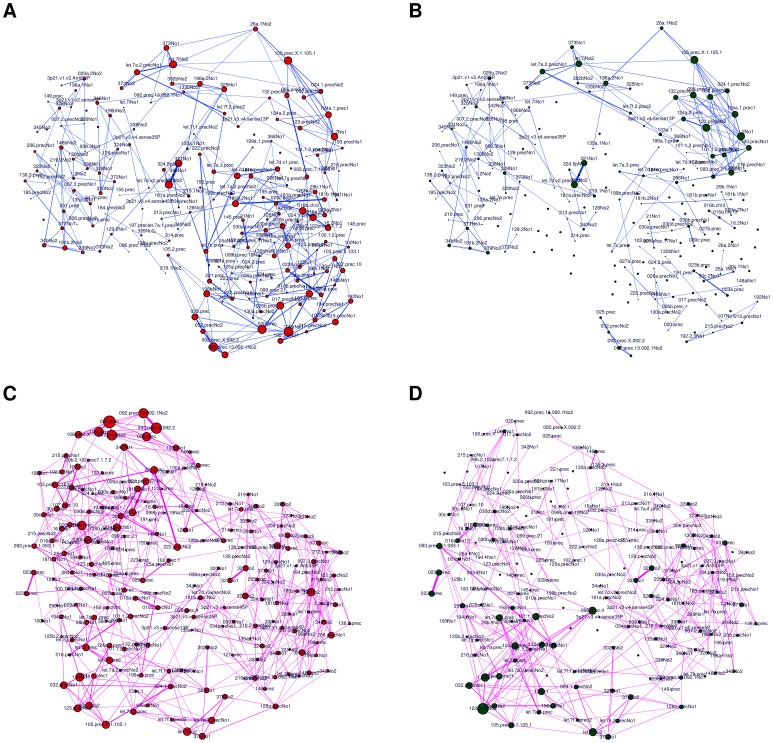
HCC-B dataset: CLR networks in the hairball representation inferred from the 4 subsets (A) Male Tumoral (MT), (B) Male non Tumoral (MnT), (C) Female Tumoral (FT), and (D) Female non Tumoral (FnT). Links are thresholded at weight 0.1, node position is fixed across the four networks, node dimension is proportional to the degree and edge width is proportional to link weight.

**Figure 16:**
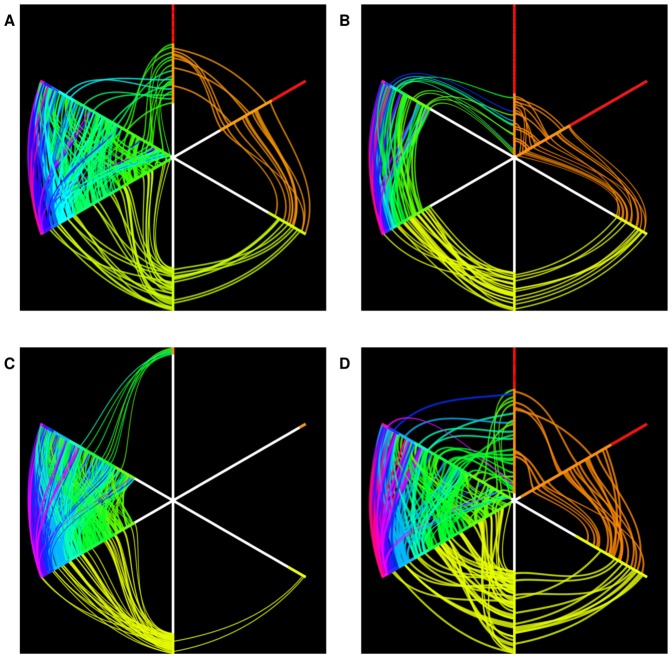
HCC-B dataset: CLR networks in the hiveplot representation inferred from the 4 subsets (A) Male Tumoral (MT), (B) Male non Tumoral (MnT), (C) Female Tumoral (FT), and (D) Female non Tumoral (FnT). Each plot consists of six axes with lines connecting points lying on the axes themselves. The axis 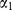 pointing upwards collects all the nodes with (unweighted) degree 0 or 1; 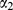, the next axis moving clockwise, is a copy of 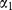; the following two axes include all nodes with degree 2, while on the remaining two axes lie all nodes with degree 3 or more. Different colors indicate different degree. Nodes on axes are ranked by degree. Lines between two consecutive axes show the network's edges and edge color is inherited by the node with smaller degree. Note the absence of links between nodes of degreee 1 and 2 in the FT case, and the smaller amount of connections between higher degree nodes in the MnT case with respect to the other three cases.

**Figure 17:**
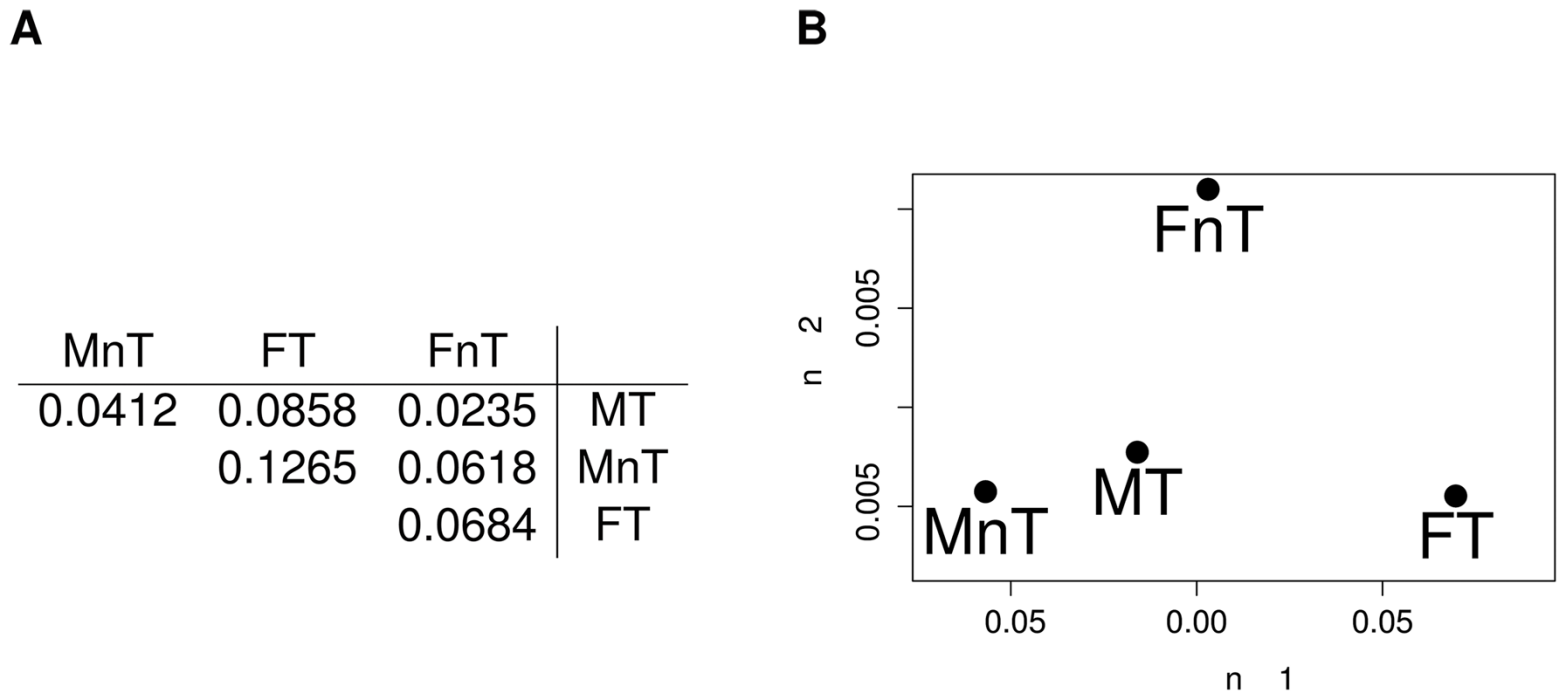
HCC-B dataset: mutual HIM distances for CLR inferred networks. Comparison of the four networks Male Tumoral (MT), Male non Tumoral (MnT), Female Tumoral (FT) and Female non Tumoral (FnT) reconstructed from the whole corresponding subsets in Tab. (A) and in the derived 2D multidimensional scaling plot (B).

Using the CLR algorithm we first generated the four networks inferred from the whole set of datasets and corresponding to the combinations of the two binary phenotypes, discarding links with weight smaller than 0.1. Two different representations of the resulting graphs are shown in Fig. 15 and Fig. 16, respectively in hairball and hiveplot layouts [59]. The second visualization technique is particularly useful in highlighting differences between networks by disaggregating the network structure according to their node degree. The four networks have different structures: more high degree links are present for tumoral tissues (graphs for MT and FT: Fig. 16 A and C) than for controls (MnT, FnT). Their density values, defined as the ratio between the number of existing edges and the maximal number of edges for the given graph), are 0.0153 (MT), 0.0092 (MnT), 0.0206 (FT) and 0.0121 (FnT). The mutual HIM distances for the four networks are reported in Fig. 17 A, together with the corresponding two-dimensional scaling plot (Fig. 17 B). The networks corresponding to the female patients (and, in particular, the FT inferred from cancer tissue) are different from those inferred for the male patients.

**Figure 18:**
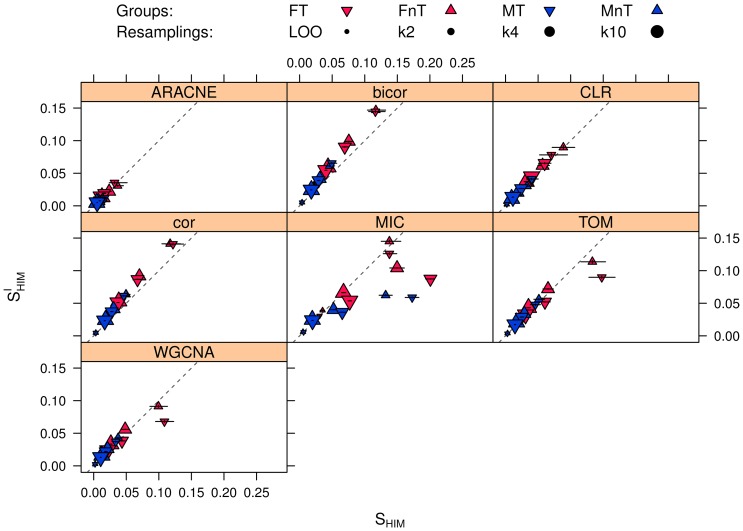
HCC-B dataset: 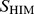 and 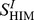 stability indicators of the four subgroups MT, MnT, FT, and FnT. The networks are inferred with six different algorithms for different values of data subsampling. MT: Male Tumoral. MnT: Male non Tumoral. FT: Female Tumoral. FnT: Female non Tumoral. Confidence intervals are represented for each experiment. Points of increasing dimension are used to represent the diverse resampling schema: Leave One Out, 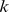-fold cross validation for 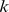 set to 2 (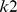), 4 (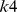) and 10 (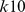) respectively.

In order to explore network reconstruction reliability, we then computed the NetSI indicators and compared the results for CLR with other six reconstruction algorithms ARACNE, cor, TOM, bicor, WGCNA and MIC. The corresponding statistics for the four subsets and different subsampling (LOO, 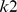, 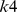, and 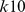) are listed in Tables 6–9 and summarized in Fig. 18. Both the resampling strategy and phenotypes have an impact on the network stability, differently for the seven methods: the networks corresponding to male patients have smaller values for 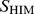 and 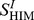 (and thus they are much more stable) than the corresponding female counterparts. The leave-one-out stability for FT and FnT is worse than for 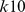 and 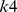 stability on MT and MnT. However phenotypes have a stronger effect than the resampling strategy. Note that while control and cancer networks display similar stability for males at all levels of subsampling ratio, the FT network is more stable than the matching FnT control networks; this is evident when the size of the subset used for inference gets smaller, in particular for 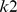.

**Table 6:** HCC-B dataset: 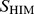 and 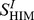 statistics for FnT network.

Algorithm	k	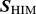	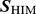 CI (min; max)	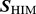 Range (min; max)	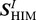	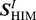 CI (min; max)	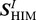 Range (min; max)
ARACNE	LOO	0.007	(0.006; 0.009)	(0.002; 0.018)	0.008	(0.008; 0.009)	(0.001; 0.045)
ARACNE	k2	0.037	(0.030; 0.045)	(0.007; 0.097)	0.032	(0.031; 0.033)	(0.002; 0.179)
ARACNE	k4	0.024	(0.021; 0.027)	(0.004; 0.060)	0.022	(0.022; 0.023)	(0.002; 0.134)
ARACNE	k10	0.013	(0.012; 0.014)	(0.003; 0.036)	0.014	(0.013; 0.014)	(0.000; 0.081)
CLR	LOO	0.022	(0.017; 0.027)	(0.003; 0.048)	0.030	(0.028; 0.032)	(0.001; 0.094)
CLR	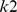	0.094	(0.071; 0.117)	(0.006; 0.257)	0.119	(0.113; 0.124)	(0.006; 0.391)
CLR	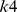	0.062	(0.054; 0.072)	(0.005; 0.203)	0.080	(0.078; 0.082)	(0.003; 0.307)
CLR	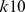	0.032	(0.029; 0.035)	(0.002; 0.093)	0.045	(0.044; 0.045)	(0.000; 0.179)
cor	LOO	0.021	(0.017; 0.026)	(0.006; 0.051)	0.031	(0.030; 0.032)	(0.002; 0.122)
cor	k2	0.117	(0.104; 0.133)	(0.059; 0.221)	0.145	(0.143; 0.147)	(0.016; 0.431)
cor	k4	0.070	(0.065; 0.078)	(0.023; 0.172)	0.093	(0.092; 0.094)	(0.008; 0.347)
cor	k10	0.038	(0.036; 0.041)	(0.014; 0.120)	0.053	(0.053; 0.054)	(0.000; 0.279)
bicor	LOO	0.026	(0.022; 0.031)	(0.012; 0.057)	0.036	(0.035; 0.037)	(0.004; 0.136)
bicor	k2	0.117	(0.105; 0.132)	(0.072; 0.227)	0.151	(0.148; 0.153)	(0.015; 0.445)
bicor	k4	0.076	(0.070; 0.083)	(0.033; 0.180)	0.100	(0.099; 0.101)	(0.007; 0.367)
bicor	k10	0.044	(0.041; 0.047)	(0.019; 0.126)	0.060	(0.059; 0.060)	(0.000; 0.286)
WGCNA	LOO	0.015	(0.013; 0.018)	(0.005; 0.033)	0.019	(0.019; 0.020)	(0.003; 0.078)
WGCNA	k2	0.099	(0.086; 0.114)	(0.035; 0.198)	0.094	(0.092; 0.096)	(0.017; 0.341)
WGCNA	k4	0.048	(0.043; 0.055)	(0.015; 0.115)	0.057	(0.056; 0.057)	(0.007; 0.251)
WGCNA	k10	0.026	(0.025; 0.028)	(0.006; 0.081)	0.033	(0.033; 0.034)	(0.000; 0.187)
MINE	LOO	0.035	(0.031; 0.039)	(0.018; 0.054)	0.040	(0.040; 0.041)	(0.003; 0.096)
MINE	k2	0.138	(0.125; 0.157)	(0.084; 0.277)	0.149	(0.146; 0.151)	(0.020; 0.482)
MINE	k4	0.150	(0.138; 0.161)	(0.050; 0.259)	0.105	(0.105; 0.106)	(0.007; 0.335)
MINE	k10	0.067	(0.064; 0.071)	(0.029; 0.138)	0.067	(0.066; 0.067)	(0.000; 0.253)
TOM	LOO	0.020	(0.017; 0.025)	(0.007; 0.047)	0.025	(0.024; 0.026)	(0.003; 0.108)
TOM	k2	0.133	(0.115; 0.154)	(0.046; 0.271)	0.117	(0.114; 0.119)	(0.011; 0.467)
TOM	k4	0.065	(0.057; 0.075)	(0.017; 0.161)	0.073	(0.072; 0.074)	(0.010; 0.348)
TOM	k10	0.035	(0.033; 0.038)	(0.007; 0.116)	0.043	(0.043; 0.044)	(0.000; 0.268)

Values of the indicators 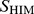 and 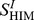 together with bootstrap confidence intervals and range for the inferred networks for different values of data subsampling.

**Table 7:** HCC-B dataset: 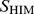 and 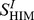 statistics for FT network.

Algorithm	k	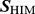	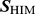 CI (min; max)	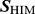 Range (min; max)	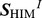	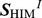 CI (min; max)	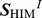 Range (min; max)
ARACNE	LOO	0.007	(0.006; 0.010)	(0.001; 0.024)	0.009	(0.009; 0.010)	(0.001; 0.053)
ARACNE	k2	0.032	(0.024; 0.053)	(0.008; 0.221)	0.036	(0.034; 0.039)	(0.003; 0.405)
ARACNE	k4	0.016	(0.014; 0.018)	(0.005; 0.054)	0.019	(0.019; 0.020)	(0.002; 0.137)
ARACNE	k10	0.012	(0.010; 0.013)	(0.002; 0.061)	0.014	(0.014; 0.014)	(0.000; 0.120)
CLR	LOO	0.022	(0.016; 0.032)	(0.002; 0.093)	0.032	(0.030; 0.035)	(0.001; 0.143)
CLR	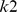	0.069	(0.056; 0.082)	(0.006; 0.154)	0.089	(0.084; 0.093)	(0.005; 0.250)
CLR	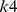	0.057	(0.049; 0.066)	(0.004; 0.190)	0.078	(0.076; 0.080)	(0.003; 0.305)
CLR	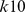	0.040	(0.037; 0.044)	(0.002; 0.177)	0.054	(0.054; 0.055)	(0.000; 0.143)
cor	LOO	0.019	(0.016; 0.024)	(0.008; 0.044)	0.028	(0.028; 0.029)	(0.003; 0.105)
cor	k2	0.122	(0.111; 0.138)	(0.079; 0.221)	0.144	(0.142; 0.147)	(0.014; 0.346)
cor	k4	0.067	(0.063; 0.073)	(0.036; 0.141)	0.088	(0.087; 0.088)	(0.007; 0.274)
cor	k10	0.037	(0.036; 0.039)	(0.019; 0.083)	0.051	(0.051; 0.051)	(0.000; 0.196)
bicor	LOO	0.022	(0.019; 0.027)	(0.012; 0.049)	0.031	(0.031; 0.032)	(0.004; 0.108)
bicor	k2	0.116	(0.107; 0.130)	(0.073; 0.217)	0.148	(0.146; 0.150)	(0.012; 0.364)
bicor	k4	0.069	(0.065; 0.074)	(0.038; 0.137)	0.092	(0.091; 0.092)	(0.009; 0.282)
bicor	k10	0.040	(0.039; 0.042)	(0.022; 0.085)	0.055	(0.055; 0.055)	(0.000; 0.202)
WGCNA	LOO	0.010	(0.008; 0.013)	(0.003; 0.025)	0.013	(0.013; 0.014)	(0.002; 0.070)
WGCNA	k2	0.109	(0.095; 0.124)	(0.026; 0.194)	0.070	(0.068; 0.072)	(0.016; 0.269)
WGCNA	k4	0.043	(0.039; 0.049)	(0.008; 0.099)	0.039	(0.038; 0.039)	(0.007; 0.182)
WGCNA	k10	0.022	(0.020; 0.023)	(0.005; 0.066)	0.024	(0.024; 0.024)	(0.000; 0.141)
MINE	LOO	0.031	(0.028; 0.034)	(0.016; 0.051)	0.032	(0.031; 0.032)	(0.003; 0.095)
MINE	k2	0.138	(0.128; 0.149)	(0.094; 0.216)	0.129	(0.128; 0.131)	(0.007; 0.321)
MINE	k4	0.201	(0.194; 0.207)	(0.132; 0.257)	0.088	(0.088; 0.089)	(0.005; 0.243)
MINE	k10	0.077	(0.075; 0.080)	(0.040; 0.138)	0.054	(0.054; 0.054)	(0.000; 0.193)
TOM	LOO	0.015	(0.011; 0.019)	(0.004; 0.038)	0.018	(0.017; 0.019)	(0.002; 0.105)
TOM	k2	0.148	(0.128; 0.170)	(0.023; 0.269)	0.092	(0.090; 0.095)	(0.012; 0.386)
TOM	k4	0.060	(0.053; 0.069)	(0.011; 0.143)	0.053	(0.052; 0.054)	(0.006; 0.272)
TOM	k10	0.031	(0.029; 0.033)	(0.006; 0.097)	0.033	(0.033; 0.033)	(0.000; 0.212)

Values of the indicators 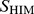 and 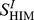 together with bootstrap confidence intervals and range for the inferred networks for different values of data subsampling.

**Table 8:** HCC-B dataset: 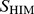 and 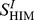 statistics for MnT network.

Algorithm	k	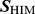	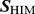 CI (min; max)	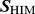 Range (min; max)	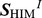	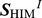 CI (min; max)	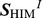 Range (min; max)
ARACNE	LOO	0.002	(0.002; 0.002)	(0.001; 0.004)	0.002	(0.002; 0.002)	(0.000; 0.011)
ARACNE	k2	0.014	(0.011; 0.016)	(0.003; 0.033)	0.012	(0.012; 0.012)	(0.002; 0.056)
ARACNE	k4	0.007	(0.006; 0.008)	(0.003; 0.022)	0.008	(0.007; 0.008)	(0.001; 0.046)
ARACNE	k10	0.005	(0.004; 0.005)	(0.002; 0.011)	0.005	(0.005; 0.005)	(0.001; 0.027)
CLR	LOO	0.002	(0.002; 0.002)	(0.000; 0.009)	0.003	(0.003; 0.003)	(0.000; 0.016)
CLR	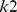	0.033	(0.026; 0.041)	(0.003; 0.104)	0.037	(0.035; 0.039)	(0.002; 0.158)
CLR	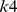	0.018	(0.015; 0.022)	(0.001; 0.067)	0.025	(0.024; 0.026)	(0.001; 0.102)
CLR	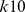	0.009	(0.008; 0.010)	(0.001; 0.034)	0.013	(0.013; 0.013)	(0.001; 0.061)
cor	LOO	0.003	(0.003; 0.004)	(0.001; 0.020)	0.005	(0.005; 0.005)	(0.000; 0.047)
cor	k2	0.050	(0.044; 0.057)	(0.028; 0.094)	0.065	(0.064; 0.066)	(0.007; 0.219)
cor	k4	0.031	(0.028; 0.034)	(0.013; 0.080)	0.042	(0.041; 0.042)	(0.005; 0.180)
cor	k10	0.018	(0.017; 0.019)	(0.007; 0.056)	0.025	(0.025; 0.025)	(0.002; 0.134)
bicor	LOO	0.004	(0.004; 0.004)	(0.001; 0.011)	0.005	(0.005; 0.005)	(0.000; 0.027)
bicor	k2	0.046	(0.041; 0.054)	(0.026; 0.103)	0.063	(0.062; 0.064)	(0.009; 0.240)
bicor	k4	0.031	(0.028; 0.034)	(0.014; 0.093)	0.042	(0.042; 0.043)	(0.005; 0.197)
bicor	k10	0.018	(0.017; 0.019)	(0.008; 0.050)	0.025	(0.024; 0.025)	(0.002; 0.122)
WGCNA	LOO	0.002	(0.002; 0.003)	(0.000; 0.019)	0.003	(0.003; 0.003)	(0.000; 0.039)
WGCNA	k2	0.037	(0.032; 0.044)	(0.009; 0.078)	0.044	(0.043; 0.045)	(0.011; 0.181)
WGCNA	k4	0.021	(0.019; 0.024)	(0.006; 0.059)	0.026	(0.026; 0.027)	(0.005; 0.132)
WGCNA	k10	0.013	(0.012; 0.014)	(0.003; 0.044)	0.016	(0.016; 0.016)	(0.002; 0.100)
MINE	LOO	0.005	(0.005; 0.005)	(0.002; 0.008)	0.006	(0.006; 0.006)	(0.000; 0.019)
MINE	k2	0.132	(0.123; 0.143)	(0.059; 0.199)	0.064	(0.063; 0.065)	(0.006; 0.233)
MINE	k4	0.052	(0.048; 0.058)	(0.021; 0.126)	0.041	(0.040; 0.041)	(0.004; 0.186)
MINE	k10	0.019	(0.019; 0.021)	(0.012; 0.055)	0.025	(0.025; 0.025)	(0.002; 0.121)
TOM	LOO	0.003	(0.003; 0.004)	(0.000; 0.023)	0.004	(0.004; 0.004)	(0.000; 0.052)
TOM	k2	0.051	(0.043; 0.060)	(0.011; 0.111)	0.058	(0.056; 0.059)	(0.010; 0.253)
TOM	k4	0.029	(0.026; 0.033)	(0.007; 0.085)	0.035	(0.034; 0.035)	(0.005; 0.193)
TOM	k10	0.017	(0.016; 0.018)	(0.003; 0.060)	0.021	(0.021; 0.021)	(0.003; 0.138)

Values of the indicators 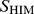 and 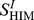 together with bootstrap confidence intervals and range for the inferred networks for different values of data subsampling.

**Table 9:** HCC-B dataset: 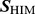 and 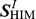 statistics for MT network.

Algorithm	k	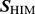	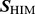 CI (min; max)	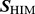 Range (min; max)	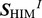	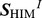 CI (min; max)	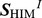 Range (min; max)
ARACNE	LOO	0.001	(0.001; 0.002)	(0.000; 0.008)	0.002	(0.002; 0.002)	(0.000; 0.017)
ARACNE	k2	0.017	(0.014; 0.020)	(0.004; 0.039)	0.012	(0.012; 0.013)	(0.002; 0.054)
ARACNE	k4	0.009	(0.008; 0.010)	(0.002; 0.021)	0.008	(0.008; 0.008)	(0.001; 0.039)
ARACNE	k10	0.005	(0.005; 0.006)	(0.001; 0.015)	0.005	(0.005; 0.005)	(0.001; 0.033)
CLR	LOO	0.002	(0.002; 0.002)	(0.000; 0.018)	0.003	(0.003; 0.003)	(0.000; 0.030)
CLR	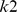	0.040	(0.033; 0.051)	(0.003; 0.146)	0.051	(0.048; 0.054)	(0.003; 0.218)
CLR	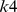	0.024	(0.020; 0.029)	(0.002; 0.099)	0.033	(0.032; 0.033)	(0.001; 0.148)
CLR	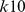	0.011	(0.010; 0.013)	(0.001; 0.048)	0.016	(0.016; 0.016)	(0.001; 0.092)
cor	LOO	0.003	(0.003; 0.004)	(0.001; 0.013)	0.005	(0.005; 0.005)	(0.000; 0.028)
cor	k2	0.046	(0.041; 0.054)	(0.029; 0.105)	0.063	(0.062; 0.064)	(0.008; 0.254)
cor	k4	0.028	(0.026; 0.030)	(0.016; 0.052)	0.038	(0.037; 0.038)	(0.005; 0.132)
cor	k10	0.017	(0.016; 0.018)	(0.008; 0.041)	0.023	(0.023; 0.023)	(0.002; 0.101)
bicor	LOO	0.004	(0.003; 0.004)	(0.001; 0.013)	0.005	(0.005; 0.005)	(0.000; 0.028)
bicor	k2	0.049	(0.044; 0.058)	(0.027; 0.112)	0.067	(0.066; 0.068)	(0.009; 0.272)
bicor	k4	0.029	(0.027; 0.031)	(0.016; 0.061)	0.039	(0.039; 0.040)	(0.004; 0.148)
bicor	k10	0.018	(0.017; 0.019)	(0.009; 0.042)	0.025	(0.025; 0.025)	(0.002; 0.105)
WGCNA	LOO	0.002	(0.002; 0.002)	(0.000; 0.009)	0.003	(0.003; 0.003)	(0.000; 0.020)
WGCNA	k2	0.034	(0.029; 0.040)	(0.009; 0.075)	0.038	(0.037; 0.039)	(0.008; 0.175)
WGCNA	k4	0.018	(0.016; 0.021)	(0.006; 0.046)	0.022	(0.022; 0.023)	(0.004; 0.122)
WGCNA	k10	0.011	(0.010; 0.012)	(0.002; 0.028)	0.013	(0.013; 0.013)	(0.002; 0.075)
MINE	LOO	0.006	(0.006; 0.006)	(0.002; 0.010)	0.006	(0.006; 0.006)	(0.000; 0.018)
MINE	k2	0.173	(0.163; 0.183)	(0.111; 0.242)	0.060	(0.059; 0.061)	(0.004; 0.220)
MINE	k4	0.065	(0.061; 0.070)	(0.027; 0.117)	0.037	(0.036; 0.037)	(0.003; 0.149)
MINE	k10	0.020	(0.019; 0.021)	(0.011; 0.046)	0.024	(0.024; 0.024)	(0.001; 0.099)
TOM	LOO	0.003	(0.003; 0.003)	(0.000; 0.012)	0.003	(0.003; 0.004)	(0.000; 0.028)
TOM	k2	0.046	(0.039; 0.054)	(0.011; 0.102)	0.049	(0.047; 0.051)	(0.008; 0.246)
TOM	k4	0.025	(0.022; 0.028)	(0.007; 0.062)	0.029	(0.029; 0.030)	(0.003; 0.168)
TOM	k10	0.015	(0.013; 0.016)	(0.003; 0.038)	0.017	(0.017; 0.017)	(0.002; 0.102)

Values of the indicators 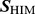 and 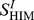 together with bootstrap confidence intervals and range for the inferred networks for different values of data subsampling.

To validate the interest of the stability analysis in terms of biological relevance and reproducibility, we applied the 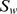 weight stability indicator and ranked accordingly the links for networks inferred with CLR on the MT, MnT, FT, FnT subsets. Our hypothesis is that, in practical cases, stability may be either an effect of real biological relevance of the link or highlight trivial relationships between nodes. In the former situation, we expect that stable links have a better chance of being reproduced on new datasets. However, the comparison between miRNA studies is often haunted by the significant changes across releases of the annotation datasets. It is not uncommon that a miRNA may get discarded or unified with others in a newer release, or that miRNA symbols may just be remapped to different sequences.

As an experiment, we considered the first six top ranked edges for the four networks, obtaining seven distinct pairs of miRNAs according to the original annotation (Tab. 10). As reported by the original GEO platform (accession number GPL4700), the HCC-B dataset [31] used the miRBase June 2004 or information collected from literature. The two miRNA hsa-mir-321No1 and hsa-mir-321No2 had the same mature Sanger registry name in the miRBase June 2004, and were eliminated and marked as tRNA in the June 2013 version. Not surprisingly they define a link (link (a) in the table) which is trivially the most stable for all four networks, regardless of the phenotypes. Similarly the pairs of miRNA defining the links (b) and (c) can be found to align to the same sequences by considering miRBase June 2013. Note that (c) is associated with the mature miR21, which is a known oncomir [60]. The hsa-mir-219-1 in link (d) is one of the 20 miRNAs in the signature proposed by Budhu and colleagues [31]; in our table it is differently stable for gender, which is consistent with being associated with estrogen regulation and overexpressed in breast cancer [61], [62]. The link (e) connects hsa-mir-326 with hsa-mir-342; the first node is known to be involved in brain tumorigenesis [63], [64] and it is directly related to microRNA gene expression profile of hepatitis C virus-associated hepatocellular carcinoma [65]. The second node is associated with different cancers and recently proposed as a diagnostic biomarker and therapeutic target for HCC [66].

**Table 10:** HCC-B dataset: evaluation of top selected miRNA–miRNA CLR links.

id	Node 1	Node 2	MT	MnT	FT	FnT
(a)	hsa-mir-321No1	hsa-mir-321No2	1	1	9	2
(b)	hsa-mir-016b.chr3	hsa-mir-16.2No1	3	12	15	309
(c)	hsa-mir-021-prec-17No1	hsa-mir-21No1	27	5	2	921
(d)	hsa-mir-219.1No1	hsa-mir-321No2	2	6	1903	314
(e)	hsa-mir-326No1	hsa-mir-342No2	132	1017	3	-
(f)	hsa-mir-192.2.3No1	hsa-mir-215.precNo1	4	300	4	3340
(g)	hsa-mir-092.prec.13.092.1No2	hsa-mir-092.prec.X.092.2	79	1158	1	404

Position in the 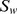 ranking for 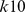 in the four cases MT, MnT, FT, and FnT.

Both nodes defining link (f) are associated with cancer [67]-[69], namely with HRC in hepatitis infection and cirrhosis [53], [70]. A search by sequence with the miRBase web-service [71] shows that the two probes hsa-mir-192.2.3No1 and hsa-mir-215.precNo have a high alignment score (e value: 0.02 [72]). In our analysis, the link is very stable in tumours and definitely unstable on controls.

Link (g) connects hsa-mir-092.prec.13.092.1No2 and hsa-mir-092.prec.X.092.2: it is the most stable for FT. The two miRNAs are to known to be associated with chronic lymphocitic leukemia [73], found respectively in genomics regions related to follicular lymphoma and advanced ovarian cancer.

Finally, we compared a disease network inferred from HCC-B [31] with the one from HCC-W using the stability indicators, also considering the 30 miRNAs signature for hepatocellular carcinoma derived from this dataset [58]. As a preliminary step, data from HCC-B were also normalized as ratios (tumor/non-tumor), then we mapped the probes from the first platform into the second with the Bowtie aligner v. 0.12.7 [74] with default parameters. A subset of 120 matching miRNAs was found, which includes 12 miRNAs from Wei's signature. Separately for each dataset, we filtered for probes having a homogeneous fold change for at least 80% of the samples, obtaining 30 miRNAs for HCC-B and 37 for HCC-W, for a total of 54 distinct miRNAs (13 were common). We inferred by CLR two disease networks 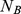 and 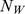 on these 54 miRNAs, and then computed the weight stability 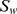 with 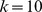 cross-validation for each network.

Within the first 10 top ranked links for 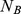, half (5) included nodes that also belonged to Wei's original signature. Moreover, the second most stable link for 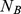 was also present in the same position for the 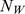 disease network, which also included 4 miRNAs from Wei's signature in its top 10 most stable edges. The common link is formed by the pair (hsa-mir-17,hsa-mir-20a), which are both strongly associated with cancer, including colorectal cancer [75], [76]. It is worth noting that hsa-mir-17 is the most strongly associated miRNA to hsa-mir-20a according to PhenomiR 2.0 [77], being at distance 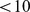 kb [71]. The same range includes hsa-mir92a-1, mapping to the hsa-mir-092.prec.13.092.1No2 probe mentioned above. A concordant overexpression of both hsa-mir-20a and hsa-mir-17 was found for this link for both the two networks 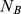 and 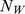.

## Conclusions

We introduced the NetSI family of indicators for assessing the variability of network reconstruction algorithms as functions of a data subsampling procedure. Our aim here is to provide the researchers with an effective tool to compare either the inference algorithms or properties of the investigated dataset. The first two indicators are global, giving a confidence measure over the whole inferred dataset and are based on a measure of distance between networks. In particular, we demonstrated the proposed framework with the HIM distance, although it is independent from the chosen metric. The other two indicators are local, associating a reliability score to the network nodes and the detected links. They are of particular interest when coupled with algorithms of proven performance, being able to capture the effect of data perturbation on the reconstruction process. We demonstrated their consistency on two synthetic datasets, testing their dependency on different inference methods, and also in comparison with the gold standards. Finally we showed an application for disease networks on miRNA hepatocellular carcinoma data; we found biological or technical evidence for the most stable links in the networks, and good reproducibility on a second dataset having relevant differences in terms of microrray technology and annotation platform.

## References

[1] Oates C, Mukherjee S (2012) Network inference and biological dynamics. Annals of Applied Statistics 6: 1209–1235. 23284600 10.1214/11-AOAS532 PMC3533376

[2] Noor A, Serpedin E, Nounou M, Nounou H, Mohamed N, et al.. (2013) An Overview of the Statistical Methods Used for Inferring Gene Regulatory Networks and Protein-Protein Interaction Networks. Advances in Bioinformatics 2013: Article ID 953814 - 12 pages. 10.1155/2013/953814 PMC3594945 23509452

[3] Zhang B, Horvath S (2005) A General Framework for Weighted Gene Co-Expression Network Analysis. Statistical Applications in Genetics and Molecular Biology 4: Article 17. 10.2202/1544-6115.1128 16646834

[4] Butte A, Tamayo P, Slonim D, Golub T, Kohane I (2000) Discovering functional relationships between RNA expression and chemotherapeutic susceptibility using relevance networks. Proceedings of the National Academy of Science 97: 12182–12186. 10.1073/pnas.220392197 PMC17315 11027309

[5] Liu Y, Qiao N, Zhu S, Su M, Sun N, et al (2013) A novel Bayesian network inference algorithm for integrative analysis of heterogeneous deep sequencing data. Cell Research 23: 440–443. 23318583 10.1038/cr.2013.8 PMC3587713

[6] Cai X, Bazerque J, Giannakis G (2013) Inference of Gene Regulatory Networks with Sparse Structural Equation Models Exploiting Genetic Perturbations. PLoS Computational Biology 9: e1003068. 23717196 10.1371/journal.pcbi.1003068 PMC3662697

[7] De Smet R, Marchal K (2010) Advantages and limitations of current network inference methods. Nature Reviews Microbiology 8: 717–729. 20805835 10.1038/nrmicro2419

[8] Kamburov A, Stelzl U, Herwig R (2012) Intscore: a web tool for confidence scoring of biological interactions. Nucleic Acids Research 40: W140–W146. 22649056 10.1093/nar/gks492 PMC3394291

[9] Feizi S, Marbach D, Médard M, Kellis M (2013) Network deconvolution as a general method to distinguish direct dependencies in networks. Nature Biotechnology 31: 726–733. 10.1038/nbt.2635 PMC3773370 23851448

[10] Phenix H, Perkins T, Kærn M (2013) Identifiability and inference of pathway motifs by epistasis analysis. Chaos 23: 025103. 23822501 10.1063/1.4807483

[11] Baralla A, Mentzen W, de la Fuente A (2009) Inferring Gene Networks: Dream or Nightmare? Annals of the New York Academy of Science 1158: 246–256. 10.1111/j.1749-6632.2008.04099.x 19348646

[12] Meyer P, Alexopoulos L, Bonk T, Califano A, Cho C, et al (2011) Verification of systems biology research in the age of collaborative competition. Nature Biotechnology 29: 811–815. 10.1038/nbt.1968 21904331

[13] He F, Balling R, Zeng AP (2009) Reverse engineering and verification of gene networks: Principles, assumptions, and limitations of present methods and future perspectives. Journal of Biotechnology 144: 190–203. 19631244 10.1016/j.jbiotec.2009.07.013

[14] Prill R, Marbach D, Saez-Rodriguez J, Sorger P, Alexopoulos L, et al (2010) Towards a Rigorous Assessment of Systems Biology Models: The DREAM3 Challenges. PLoS ONE 5: e9202. 20186320 10.1371/journal.pone.0009202 PMC2826397

[15] Logsdon B, Mezey J (2010) Gene Expression Network Reconstruction by Convex Feature Selection when Incorporating Genetic Perturbations. PLoS Computational Biology 6: e1001014. 21152011 10.1371/journal.pcbi.1001014 PMC2996324

[16] Gillis J, Pavlidis P (2011) The role of indirect connections in gene networks in predicting function. Bioinformatics 27: 1860–1866. 21551147 10.1093/bioinformatics/btr288 PMC3117376

[17] Miller M, Feng XJ, Li G, Rabitz H (2012) Identifying Biological Network Structure, Predicting Network Behavior, and Classifying Network State With High Dimensional Model Representation (HDMR). PLoS ONE 7: e37664. 22723838 10.1371/journal.pone.0037664 PMC3377689

[18] Altay G (2012) Empirically determining the sample size for large-scale gene network inference algorithms. IET Systems Biology 6: 35–43. 22519356 10.1049/iet-syb.2010.0091

[19] Szederkenyi G, Banga J, Alonso A (2011) Inference of complex biological networks: distinguishability issues and optimization-based solutions. BMC Systems Biology 5: 177. 22034917 10.1186/1752-0509-5-177 PMC3305990

[20] Altay G, Emmert-Streib F (2010) Revealing differences in gene network inference algorithms on the network level by ensemble methods. Bioinformatics 26: 1738–1744. 20501553 10.1093/bioinformatics/btq259

[21] Krishnan A, Giuliani A, Tomita M (2007) Indeterminacy of Reverse Engineering of Gene Regulatory Networks: The Curse of Gene Elasticity. PLoS ONE 2: e562. 17593963 10.1371/journal.pone.0000562 PMC1894653

[22] Madhamshettiwar P, Maetschke S, Davis M, Reverter A, Ragan M (2012) Gene regulatory network inference: evaluation and application to ovarian cancer allows the prioritization of drug targets. Genome Medicine 4: 41. 22548828 10.1186/gm340 PMC3506907

[23] Marbach D, Prill RJ, Schaffter T, Mattiussi C, Floreano D, et al (2010) Revealing strengths and weaknesses of methods for gene network inference. Proceedings of the National Academy of Science 107: 6286–6291. 10.1073/pnas.0913357107 PMC2851985 20308593

[24] Marbach D, Costello J, Kuffner R, Vega N, Prill R, et al (2012) Wisdom of crowds for robust gene network inference. Nature Methods 9: 796–804. 22796662 10.1038/nmeth.2016 PMC3512113

[25] Davison A, Hinkley D (1997) Bootstrap Methods and Their Applications. Cambridge University Press.

[26] Jurman G, Visintainer R, Riccadonna S, Filosi M, Furlanello C (2013) The HIM glocal metric and kernel for network comparison and classification. ArXiv:1201.2931 [math.CO].

[27] Barla A, Jurman G, Visintainer R, Squillario M, Filosi M, et al.. (2013) A Machine Learning Pipeline for Discriminant Pathways Identification. In: Kasabov N, editor, Springer Handbook of Bio-/Neuroinformatics, Berlin: Springer Verlag, chapter 53. p. 1200.

[28] Margolin A, Nemenman I, Basso K, Wiggins C, Stolovitzky G, et al (2006) ARACNE: an algorithm for the reconstruction of gene regulatory networks in a mammalian cellular context. BMC Bioinformatics 7: S7. 10.1186/1471-2105-7-S1-S7 PMC1810318 16723010

[29] Faith J, Hayete B, Thaden J, Mogno I, Wierzbowski J, et al (2007) Large-Scale Mapping and Validation of Escherichia coli Transcriptional Regulation from a Compendium of Expression Profiles. PLoS Biology 5: e8. 17214507 10.1371/journal.pbio.0050008 PMC1764438

[30] Horvath S (2011) Weighted Network Analysis: Applications in Genomics and Systems Biology. Springer.

[31] Budhu A, Jia HL, Forgues M, Liu CG, Goldstein D, et al (2008) Identification of Metastasis-Related MicroRNAs in Hepatocellular Carcinoma. Hepatology 47: 897–907. 18176954 10.1002/hep.22160

[32] Ji J, Shi J, Budhu A, Yu Z, Forgues M, et al (2009) MicroRNA Expression, Survival, and Response to Interferon in Liver Cancer. New England Journal of Medicine 361: 1437–1447. 19812400 10.1056/NEJMoa0901282 PMC2786938

[33] Visintainer R (2012) Distances and Stability in Biological Network Theory. Ph.D. thesis, DISI, University of Trento.

[34] Tun K, Dhar P, Palumbo M, Giuliani A (2006) Metabolic pathways variability and sequence/ networks comparisons. BMC Bioinformatics 7: 24. 16420696 10.1186/1471-2105-7-24 PMC1360688

[35] Dougherty E (2010) Validation of gene regulatory networks: scientific and inferential. Briefings in Bioinformatics 12: 245–252. 21183477 10.1093/bib/bbq078

[36] Ipsen M, Mikhailov A (2002) Evolutionary reconstruction of networks. Physical Review E 66: 046109. 10.1103/PhysRevE.66.046109 12443261

[37] Jurman G, Visintainer R, Furlanello C (2011) An introduction to spectral distances in networks. Frontiers in Artificial Intelligence and Applications 226: 227–234.

[38] Fay D, Moore A, Filosi M, Jurman G (2013) Graph metrics as summary statistics for Approximate Bayesian Computation with application to network model parameter estimation. In press.

[39] Chung F (1997) Spectral Graph Theory. American Mathematical Society.

[40] Spielman D (2009) Spectral Graph Theory: The Laplacian (Lecture 2). Lecture notes.

[41] Tönjes R, Blasius B (2009) Perturbation Analysis of Complete Synchronization in Networks of Phase Oscillators. Physical Review E 80: 026202. 10.1103/PhysRevE.80.026202 19792226

[42] Atay F, Bıyıkoğlu T, Jost J (2006) Network synchronization: Spectral versus statistical properties. Physica D Nonlinear Phenomena 224: 35–41.

[43] Langfelder P, Horvath S (2008) WGCNA: an R package for weighted correlation network analysis. BMC Bioinformatics 9: 559. 19114008 10.1186/1471-2105-9-559 PMC2631488

[44] Song L, Langfelder P, Horvath S (2012) Comparison of co-expression measures: mutual information, correlation, and model based indices. BMC Bioinformatics 13: 328. 23217028 10.1186/1471-2105-13-328 PMC3586947

[45] Reshef D, Reshef Y, Finucane H, Grossman S, McVean G, et al (2011) Detecting novel associations in large datasets. Science 6062: 1518–1524. 10.1126/science.1205438 PMC3325791 22174245

[46] Speed T (2011) A Correlation for the 21st Century. Science 6062: 1502–1503. 10.1126/science.1215894 22174235

[47] Nature Biotechnology (2012) Finding correlations in big data. Nature Biotechnology 30: 334–335. 10.1038/nbt.2182 22491290

[48] Albanese D, Filosi M, Visintainer R, Riccadonna S, Jurman G, et al (2013) minerva and minepy: a C engine for the MINE suite and its R, Python and MATLAB wrappers. Bioinformatics 29: 407–408. 23242262 10.1093/bioinformatics/bts707

[49] Ambroise J, Robert A, Macq B, Gala JL (2012) Transcriptional Network Inference from Functional Similarity and Expression Data: A Global Supervised Approach. Statistical Applications in Genetics and Molecular Biology 11: Article 2. 10.2202/1544-6115.1695 22499684

[50] Schaffter T, Marbach D, Floreano D (2011) GeneNetWeaver: in silico benchmark generation and performance profiling of network inference methods. Bioinformatics 27: 2263–2270. 21697125 10.1093/bioinformatics/btr373

[51] Jiao Y, Lawler K, Patel G, Purushotham A, Jones A, et al (2011) DART: Denoising Algorithm based on Relevance network Topology improves molecular pathway activity inference. BMC Bioinformatics 12: 403. 22011170 10.1186/1471-2105-12-403 PMC3228554

[52] Allen J, Xie Y, Chen M, Girard L, Xiao G (2012) Comparing Statistical Methods for Constructing Large Scale Gene Networks. PLoS ONE 7: e29348. 22272232 10.1371/journal.pone.0029348 PMC3260142

[53] Volinia S, Galasso M, Costinean S, Tagliavini L, Gamberoni G, et al (2010) Reprogramming of miRNA networks in cancer and leukemia. Genome Research 20: 589–599. 20439436 10.1101/gr.098046.109 PMC2860161

[54] Bandyopadhyay S, Mitra R, Maulik U, Zhang M (2010) Development of the human cancer microRNA network. Silence 1: 6. 20226080 10.1186/1758-907X-1-6 PMC2835996

[55] Law PTY, Wong N (2011) Emerging roles of microRNA in the intracellular signaling networks of hepatocellular carcinoma. Journal of Gastroenterology and Hepatology 26: 437–449. 21332540 10.1111/j.1440-1746.2010.06512.x

[56] Gu Z, Zhang C, Wang J (2012) Gene regulation is governed by a core network in hepatocellular carcinoma. BMC Systems Biology 6: 32. 22548756 10.1186/1752-0509-6-32 PMC3403900

[57] Troyanskaya O, Cantor M, Sherlock G, Brown P, Hastie T, et al (2001) Missing value estimation methods for DNA microarrays. Bioinformatics 17: 520–525. 11395428 10.1093/bioinformatics/17.6.520

[58] Wei RR, Huang GL, Zhang MY, Li BK, Zhang HZ, et al (2013) Clinical significance and prognostic value of microRNA expression signatures in hepatocellular carcinoma. Clinical Cancer Research 19: 4780–4791. 23812667 10.1158/1078-0432.CCR-12-2728

[59] Krzywinski M, Birol I, Jones S, Marra M (2012) Hive plots-rational approach to visualizing networks. Briefings in Bioinformatics 13: 627–644. 22155641 10.1093/bib/bbr069

[60] Asangani I, Rasheed S, Nikolova D, Leupold J, Colburn N, et al (2008) MicroRNA-21 (miR-21) post-transcriptionally downregulates tumor suppressor Pdcd4 and stimulates invasion, intravasation and metastasis in colorectal cancer. Oncogene 27: 2128–2136. 17968323 10.1038/sj.onc.1210856

[61] Mellios N, Galdzicka M, Ginns E, Baker S, Rogaev E, et al (2012) Gender-Specific Reduction of Estrogen-Sensitive Small RNA, miR-30b, in Subjects With Schizophrenia. Schizophrenia Bulletin 38: 433–443. 20732949 10.1093/schbul/sbq091 PMC3329977

[62] Zhang L, Huang J, Yang N, Greshock J, Megraw M, et al (2006) microRNAs exhibit high frequency genomic alterations in human cancer. Proceedings of the National Academy of Science 103: 9136–9141. 10.1073/pnas.0508889103 PMC1474008 16754881

[63] Qiu S, Lin S, Hu D, Feng Y, Tan Y, et al (2013) Interactions of miR-323/miR-326/miR-329 and miR-130a/miR-155/miR-210 as prognostic indicators for clinical outcome of glioblastoma patients. Journal of Translational Medicine 11: 10. 23302469 10.1186/1479-5876-11-10 PMC3551827

[64] Kefas B, Comeau L, Floyd D, Seleverstov O, Godlewski J, et al (2009) The Neuronal MicroRNA miR-326 Acts in a Feedback Loop with Notch and Has Therapeutic Potential against Brain Tumors. The Journal of Neuroscience 29: 15161–15168. 19955368 10.1523/JNEUROSCI.4966-09.2009 PMC2823067

[65] Varnholt H, Drebber U, Schulze F, Wedemeyer I, Schirmacher P, et al (2008) MicroRNA gene expression profile of hepatitis C virus-associated hepatocellular carcinoma. Hepatology 47: 1223–1232. 18307259 10.1002/hep.22158

[66] Li X, Yang W, Lou L, Chen Y, Wu S, et al (2014) microRNA: A Promising Diagnostic Biomarker and Therapeutic Target for Hepatocellular Carcinoma. Digestive Diseases and Sciences January 2014: 1–9. 10.1007/s10620-013-3006-1 24390674

[67] Braun C, Zhang X, Savelyeva I, Wolff S, Moll U, et al (2008) p53-Responsive MicroRNAs 192 and 215 Are Capable of Inducing Cell Cycle Arrest. Cancer Research 68: 10094–10104. 19074875 10.1158/0008-5472.CAN-08-1569 PMC2836584

[68] Georges S, Biery M, Kim S, Schelter J, Guo J, et al (2008) Coordinated Regulation of Cell Cycle Transcripts by p53-Inducible microRNAs, miR-192 and miR-215. Cancer Research 68: 10105–10112. 19074876 10.1158/0008-5472.CAN-08-1846

[69] Pichiorri F, Suh SS, Rocci A, Luca LD, Taccioli C, et al (2010) Downregulation of p53-inducible microRNAs 192, 194, and 215 Impairs the p53/MDM2 Autoregulatory Loop in Multiple Myeloma Development. Cancer Cell 18: 367–381. 20951946 10.1016/j.ccr.2010.09.005 PMC3561766

[70] Jiang J, Gusev Y, Aderca I, Mettler T, Nagorney D, et al (2008) Association of MicroRNA Expression in Hepatocellular Carcinomas with Hepatitis Infection, Cirrhosis, and Patient Survival. Clinical Cancer Research 14: 419–427. 18223217 10.1158/1078-0432.CCR-07-0523 PMC2755230

[71] Kozomara A, Griffiths-Jones S (2011) miRBase: integrating microRNA annotation and deepsequencing data. Nucleic Acids Research 39: D152–D157. 21037258 10.1093/nar/gkq1027 PMC3013655

[72] Altschul SF, Gish W, Miller W, Myers EW, Lipman DJ (1990) Basic local alignment search tool. Journal of Molecular Biology 215: 403–410. 2231712 10.1016/S0022-2836(05)80360-2

[73] Calin G, Liu CG, Sevignani C, Ferracin M, Felli N, et al (2004) MicroRNA profiling reveals distinct signatures in B cell chronic lymphocytic leukemias. Proceedings of the National Academy of Sciences 101: 11755–11760. 10.1073/pnas.0404432101 PMC511048 15284443

[74] Langmead B, Trapnell C, Pop M, Salzberg S (2009) Ultrafast and memory-efficient alignment of short DNA sequences to the human genome. Genome Biology 10: R25. 19261174 10.1186/gb-2009-10-3-r25 PMC2690996

[75] Murakami Y, Yasuda T, Saigo K, Urashima T, Toyoda H, et al (2005) Comprehensive analysis of microRNA expression patterns in hepatocellular carcinoma and non-tumorous tissues. Oncogene 25: 2537–2545. 10.1038/sj.onc.1209283 16331254

[76] Connolly E, Melegari M, Landgraf P, Tchaikovskaya T, Tennant BC, et al.. (2008) Elevated expression of the mir-17-92 polycistron and mir-21 in hepadnavirus-associated hepatocellular carcinoma contributes to the malignant phenotype. The American Journal of Pathology 173: 856 – 864. 10.2353/ajpath.2008.080096 PMC2527078 18688024

[77] Ruepp A, Kowarsch A, Schmidl D, Buggenthin F, Brauner B, et al (2010) PhenomiR: a knowledgebase for microRNA expression in diseases and biological processes. Genome Biology 11: R6. 20089154 10.1186/gb-2010-11-1-r6 PMC2847718

